# A Data-Driven Synthesis of Research Evidence for Domains of Hearing Loss, as Reported by Adults With Hearing Loss and Their Communication Partners

**DOI:** 10.1177/2331216517734088

**Published:** 2017-10-05

**Authors:** Venessa Vas, Michael A. Akeroyd, Deborah A. Hall

**Affiliations:** 1 National Institute for Health Research (NIHR) Nottingham Biomedical Research Centre, Nottingham, UK; 2 Otology and Hearing Group, Division of Clinical Neuroscience, School of Medicine, 170718 University of Nottingham, UK; 3 Medical Research Council Institute of Hearing Research, School of Medicine, The 170718 University of Nottingham, University Park, Nottingham, UK

**Keywords:** audiology, adult otolaryngology, adult aural rehabilitation, hearing interventions, hearing aids, patient complaints, patient-report, outcome measures

## Abstract

A number of assessment tools exist to evaluate the impact of hearing loss, with little consensus among researchers as to either preference or psychometric adequacy. The item content of hearing loss assessment tools should seek to capture the impact of hearing loss on everyday life, but to date no one has synthesized the range of hearing loss complaints from the perspectives of the person with hearing loss and their communication partner. The current review aims to synthesize the evidence on person with hearing loss- and communication partner-reported complaints of hearing loss. Searches were conducted in Cos Conference Papers Index, the Cumulative Index to Nursing and Allied Health Literature, Excerpta Medica Database, PubMed, Web of Science, and Google Scholar to identify publications from May 1982 to August 2015. A manual search of four relevant journals updated the search to May 2017. Of the 9,516 titles identified, 78 records (comprising 20,306 participants) met inclusion criteria and were taken through to data collection. Data were analyzed using meta-ethnography to form domains representing the person with hearing loss- and communication partner-reported complaints of hearing loss as reported in research. Domains and subdomains mutual to both perspectives are related to “Auditory” (listening, communicating, and speaking), “Social” (relationships, isolation, social life, occupational, and interventions), and “Self” (effort and fatigue, emotions, identity, and stigma). Our framework contributes fundamental new knowledge and a unique resource that enables researchers and clinicians to consider the broader impacts of hearing loss. Our findings can also be used to guide questions during diagnostic assessment and to evaluate existing measures of hearing loss.

## Introduction

Hearing loss affects about 300 million adults worldwide (World Health Organization, 2015), and there is general consensus that hearing loss can have a negative impact on various aspects of an individual’s quality of life. Difficulties in everyday life attributed to hearing loss vary considerably from person to person, and the degree of difficulties correlates poorly with audiometric profiles (Gatehouse & Noble, 2004). Aspects of life affected by hearing loss in relation to hearing aid uptake, such as personality, cannot be measured using performance-based technical measures (Cox, 2003), and other effects that individuals with hearing loss may experience are in activity limitations or participation restrictions (Helvik et al., 2006), which too cannot be measured directly in the clinic. Patient report is recommended as the most appropriate measure for capturing a representative account of hearing-related complaints (Macefield et al., 2014). The diversity of generic and hearing-specific complaints such as hearing disability, hearing handicap, quality of life, hearing aid benefit, communication, and psychological outcomes perhaps helps to explain why so many questionnaires have been developed to explore the impact of hearing loss. One survey found 140 questionnaires, with about one third of all their collated items being concerned with the person’s own hearing, another third with the impact of it, and a quarter with hearing aids (Akeroyd, Wright-Whyte, Holman, & Whitmer, 2015; Whitmer, Wright-Whyte, Holman, & Akeroyd, 2016). A systematic review of audiological research (Granberg, Dahlstrom, Moller, Kahari, & Danermark, 2014) identified 39 different standardized patient-reported measures in use. The Hearing Handicap Inventory for the Elderly (HHIE; Ventry & Weinstein, 1982) was the most common questionnaire but was used just seven times out of a total of 122 articles, and the Abbreviated Profile of Hearing Aid Benefit (Cox & Alexander, 1995) was the second most common, being used just four times. The frequency of usage of individual instruments is therefore low. This pattern of “many questionnaires in use, yet little consensus” (Granberg, Dahlstrom, et al., 2014) has been confirmed more recently by a scoping review (Barker, MacKenzie, Elliott, & de Lusignan, 2015). Some researchers have developed hearing loss assessment questionnaires that tap into specific domains, such as the social and emotional consequences of hearing loss that are in the HHIE. Other researchers have opted to develop questionnaires that allow for the person with hearing impairment (PHI) to indicate important aspects of their life affected by hearing loss, such as the Glasgow Profile of Hearing Aid Benefit (Gatehouse, 1999). Perhaps the most promising comprehensive project here comes from the International Classification of Functioning, Disability and Health (ICF); see details below. This issue is also represented by the vast number of studies that have investigated the negative consequences of hearing loss that span beyond auditory impairment (Arlinger, 2003; Barker, Leighton, & Ferguson, 2017).

It is also desirable that the items in questionnaires are fully determined by patient input, yet how items in many published questionnaires were selected is not always reported clearly in terms of either stakeholder input (professionals, patients or both) or methods used to collect potential content information. However, without this knowledge, we cannot be sure that examining questionnaire items or subscales in isolation will give the *full* range of patient-reported domains relating to the everyday impact of hearing loss. For example, of the nine questionnaires developed to measure the impact of hearing loss on day-to-day life, only five explicitly involved patient involvement using qualitative methods, namely the Communication Profile for the Hearing Impaired (Demorest & Erdman, 1987), Performance Inventory for Profound and Severe Loss (Owens & Raggio, 1988), Satisfaction with Amplification in Daily Life (Cox & Alexander, 1999), the Speech, Spatial and Qualities of Hearing Scale (Gatehouse & Noble, 2004), and the International Outcome Inventory for Hearing Aids (Cox & Alexander, 2002). The remainder reported that clinicians generated questions based on clinical experience (HHIE [Ventry & Weinstein, 1982] and Hearing Aid Performance Inventory [Walden, Demorest, & Hepler, 1984]) or based on modifying existing questionnaires (the Abbreviated Profile of Hearing Aid Benefit [Cox & Alexander, 1995] and Glasgow Benefit Inventory [Robinson, Gatehouse, & Browning, 1996]).

In 2001, the World Health Assembly endorsed the ICF for use as an international standard for describing and measuring health and disability (WHO, 2001). It offers a model that integrates biological, psychological, and social aspects of human functioning, aiming to integrate patient and professional perspectives to create a comprehensive list of categories relevant to adult hearing loss (Danermark, Granberg, Kramer, Selb, & Moller, 2013). Many groups across health conditions have used the ICF to develop a “Core Set” which consists of a comprehensive list of categories that are of particular relevance to a specific condition. In 2008, an international working group supported by WHO generated a Core Set for adult hearing loss (Danermark et al., 2013). The aim was to identify which areas of functioning, disability, and environment were considered important from the perspective of adults with hearing loss, using seven open questions. The questions used to elicit information from participants were developed specifically to address the different components of the ICF framework describing the person’s level of functioning with hearing loss. The brief ICF Core Set for hearing loss has 27 domains within four category labels: body functions, body structures, participation, and environmental factors. “Body functions” describes the physiological functioning of the body, “Activities” refer to “the execution of a task” and “participation” refers to “involvement in life situations” (Granberg, Dahlstrom, et al., 2014; Granberg, Pronk, et al., 2014). “Environmental factors” comprises the environmental factors such as the physical and social environment in which people live their lives (Granberg, Dahlstrom, et al., 2014; Granberg, Pronk, et al., 2014). However, any patient-reported symptom that fell outside the ICF framework were excluded, and so even in this, essential symptoms may not be reflected in the Core Sets for hearing loss.

Further, hearing loss affects not only the individual but also those close to them (Kamil & Lin, 2015). This group of people are often called “communication partners” (Manchaiah & Stephens, 2012). The term *communication partners* is here taken as referring to “those with whom the person with hearing impairment communicates with on a regular basis … their spouse, siblings, children, friends, relatives, colleagues, and carers” (Manchaiah, Stephens, Zhao, & Kramer, 2012, p. 1). Two reviews are already available of the growing body of literature on the impact of hearing loss on communication partners. One identified 24 articles relating to the impact of hearing loss on the communication partner (Kamil & Lin, 2015). This systematic review included observational clinical studies, randomized clinical trials, and epidemiologic studies, and so the focus of data synthesis was based on outcome metrics and study findings, more so than on self-reported experiences. The authors identified social life, burden of communication, and quality of life as emerging dimensions of generic and hearing-specific complaints; they also identified a gap in knowledge about the effects on the communication partner’s mental health. The review also focussed on the quality of life and mental health of communication partners. However, the authors did not synthesize studies that explored the impact on those experiencing or diagnosed with hearing loss. Baker et al. (2017) conducted a metasynthesis of qualitative literature that explored the evidence for the psychosocial implications of hearing loss for people with hearing loss and their communication partners. The authors identified four overarching themes: the effect of hearing loss, the response to hearing(s), stigma and identity, and coping strategies (Barker et al., 2017).

To date, there has been no comprehensive synthesis of what patients *and* their communication partners themselves report is the impact of hearing loss on them. Accordingly, the present review is novel in that its first primary objective is to collect and synthesize generic and hearing-specific complaints in everyday life that are reported by people with hearing loss and their communication partners. We generated two frameworks summarizing the data about living with hearing loss: (a) the personal impact of hearing loss from the perspective of the PHI (termed the Domains of Hearing Loss-person with hearing loss, “DoHL-P,” framework) and (b) the impact hearing loss from the perspective of communication partners (“DoHL-CP” framework). These frameworks consist of a hierarchical framework with supradomain, domain, and subdomain groupings, all using inductive (data-driven) methods. A “domain” refers to a broad area of life that is negatively affected by hearing loss (e.g., hearing sounds; see Results section for details). A “subdomain” refers to a distinct aspect of life that is affected by hearing loss such as a particular situation or scenario (e.g., hearing telephone ring). A “supra-domain” aims to broadly categorize the domains (e.g., auditory). Subsequently, we then sought to (a) identify similarities and differences in the evidence collected from people with hearing loss and from communication partner(s), (b) compare the DoHL-P with the ICF Core Set for hearing loss, and (c) investigate whether any domains or subdomains vary as a function of hearing loss severity (data permitting).

## Materials and Methods

We followed the search strategy, data collection and synthesis methods, and the quality assessment as laid out in a predefined protocol (Vas, Akeroyd, & Hall, 2016). Note that the study is now considered to be a synthesis of the research evidence rather than a systematic review. This is because the heterogeneity of the included studies in terms of qualitative and quantitative data collection, prevented meta-analysis and risk of bias assessment (key components of a systematic review) from being carried out.

### Inclusion Criteria

We searched for studies that have reported what adults with hearing loss and communication partners report as problematic in everyday life. To be eligible for inclusion in the review, studies must have recruited adults (men and/or women) ≥ 18 years old who had been diagnosed with mild-to-profound hearing loss as the primary condition of interest or communication partner(s) who could be of any age or hearing status. Participants were required to use oral communication as their primary mode of communication, but there was no restriction to those people using hearing aids or other assistive listening devices. Any studies that investigated the perspective of professionals only regarding the impact of hearing loss were excluded because it was not in the scope of our research question.

We included intervention studies where data in these studies were taken at the initial assessment, as well as non-intervention studies. There was no restriction on the type of study design. Resource and language limitations within the team led us to limit studies to those published in the English language. The search was limited to publications on or after May 1, 1982, because the HHIE questionnaire (Ventry & Weinstein, 1982) was published then. Eligible publications were journal articles, book chapters, and conference proceedings that reported interventions, observational or cross-sectional studies, and those that employed questionnaires, interviews, or focus groups to collect data relating to our primary question, but case reports, articles for professional magazines, and web-based discussion forums were excluded. Published systematic reviews were not subject to the data collection process itself, but their reference lists were manually searched to identify any additional eligible studies. There were no restrictions on research settings.

### Information Sources

To support an exhaustive literature search, published articles were identified through numerous electronic databases: Cos Conference Papers Index, the Cumulative Index to Nursing and Allied Health Literature, Excerpta Medica Database, PubMed (including MEDLINE), and Web of Science. Google Scholar was also searched page by page until it contained no relevant articles. All electronic searches were conducted on August 31, 2015. Finally, to ensure that the review was up-to-date, we conducted a manual search of the top four journals in which eligible studies had been sourced (i.e., *Ear and Hearing*, *International Journal of Audiology*, *Audiology*, and *Journal of the American Academy of Audiology*) from August 2015 to April 2017. This final manual search was conducted on May 3, 2017.

### Search Strategy

The electronic database search required “hearing” in the title or abstract, in conjunction with additional relevant search times in the title or abstract. The search strategy was reported in the protocol (Vas, Akeroyd, & Hall, 2016), but in brief, the search terms were as follows: (a) hearing AND problem OR complain* OR symptom OR impairment OR difficult* OR concern* OR impact AND (b) patient OR communication partner OR partner OR spouse OR significant (other) OR famil*. The search strategy was modified to accommodate to the settings of each database and where possible was limited to humans, adult, English language, and post-May 1982.

### Study Selection

Study selection commenced once searches of the preselected databases were conducted, and it consisted of three stages: title screening, abstract screening, and full-text review. First, all of the studies derived from each database search were screened for inclusion by one researcher (VV). Studies that were evidently irrelevant to the eligibility criteria of the systematic review based on the title were excluded. Next, the abstracts of studies that passed the title screen were independently screened in an unblinded standardized manner by two reviewers (DAH and VV). The reviewers screened the abstracts according to the eligibility criteria of the review, such as the objectives, methods, and language of the study. Disagreements between reviewers were resolved through discussion. The full text of studies that met the eligibility criteria based on the abstract or where there was uncertainty were obtained for review. The full text of studies were then independently screened by the same two reviewers according to the eligibility criteria. Those that met inclusion of the review were retained for data collection.

### Data Collection Process

Data collection was conducted using a prespecified electronic data collection form. To minimize observer bias, guidance material was created prior to the data collection process (see Supplementary file A), and then the data collection form and guidance were both piloted and revised across three iterations by VV and DAH. VV carried out data collection and consulted with DAH and MAA to resolve any uncertainties.

### Data Items

For each included study, we recorded the researcher performing data collection, study authors, title, year of publication, type of publication (e.g., journal article, book chapter, or conference paper), and country of origin. For the study characteristics, we recorded the study design, whether or not hearing loss was the primary condition of interest, the wording of questions (open, closed, or open and closed), sample size, and theoretical framework reported by authors (if any). For the data items relating to participant characteristics, we recorded their mean age, gender, setting (e.g., academic, clinical), and hearing status (including mean audiometric thresholds, description of hearing loss severity, or etiology of hearing loss).

For the complaints reported by both the PHI and their communication partners, we recorded the measure used to obtain each hearing loss complaint or domain (questionnaire, interview, or focus group), the domain as described in the text, author examples or participant quotes describing their complaints, and perspective (referring to self or to other). For studies using closed-set questionnaires to assess the impact of hearing loss, we extracted data only for those subscales or questionnaire items that had been highlighted by the study findings or conclusions as reflecting experienced complaints (i.e., we did not simply extract data indiscriminately on all subscales or items of a questionnaire). For intervention studies, data pertaining to our research question was only extracted at the initial assessment and therefore we did not extract information about effectiveness of treatments. Given that our primary research question was to identify what are the reported complaints in everyday life experienced by adults with hearing loss as well as their communication partners, our data collection carefully considered those complaints, examples, and quotes given by each party in terms of how hearing loss affected them personally. The terminology used by study authors, in the form of reported examples or quotes, was important to help us understand each authors’ epistemological frame and hence to interpret their concept of each domain.

### Synthesis of Results

Reported complaints, examples, and quotes associated with hearing loss typically referred to the negative functional impact on hearing ability or other psychosocial consequences of hearing loss, but examples were wide ranging. The aim of the data synthesis was to identify and group together similar data characteristics across studies into domains, and so data synthesis used a meta-ethnographic approach (Campbell et al., 2011; Noblit & Hare, 1988). Meta-ethnography aims to identify commonality across studies allowing for themes to emerge from the qualitative data. It thus utilizes an inductive approach, resulting in a reconceptualization of the data, and so is appropriate for synthesizing the qualitative data extracted from the included publications. Data synthesis was guided by Noblit’s (Noblit & Hare, 1988) method.

#### Getting started

The specific research question that data synthesis aimed to address was to collect and synthesize generic and hearing-specific complaints in everyday life that are reported by people with hearing loss and also by communication partners.

#### Deciding what is relevant to the initial interest

The scope of the synthesis was to focus on what studies had reported the personal impact of hearing loss on individuals with hearing loss and the personal impact of hearing loss on communication partners. Studies included in the data synthesis were assumed to be of acceptable quality in terms of methods and reliability of results.

#### Reading the studies

Familiarization of the studies was first conducted in the study screening stage of the abstracts and full text of the studies. Data pertaining to the research question was extracted for data synthesis during the data collection process.

#### Determining how the studies are related

The first step of synthesizing the data required searching for and grouping the domain data under descriptive labels that contained recurring keywords, such as “stigma” and “withdrawal.” All of the extracted complaints and domains were printed onto card for analysis by the research team. To clearly identify which complaints were comparable, the printed cards were sorted into groups, then the researchers looked through the cards for common and recurring themes. The extracted qualitative data were synthesized at domain level in the first instance. Preliminary domain groupings emerged from the given words, phrases, and sentences taken directly from the full texts (without any abstraction). Complaints that appeared to be nonspecific (e.g., “background noise” or “domestic life”) or contained limited information (e.g., “public incidents” or “dependence”) were temporarily placed in a “miscellaneous” group and carried forward to further review. Individual data items that could not be consolidated into the final domain grouping framework are reported as Supplementary file (available in Supplementary file B).

#### Translating the studies into one another

The preliminary groupings were then thoroughly reviewed by the research team. The descriptive labels used to name each grouping were also reviewed based on the revised domain keywords. Suggestions were shared among all three authors leading to a harmonization of the domain classification (Saldaña, 2015).

#### Synthesizing translations

At this stage, not only did we refine the domain groupings and their descriptive labels, but we also created supra-domains (termed *Auditory*, *Social*, *Self*) at a higher level of abstraction (Campbell et al., 2003), and subdomains at a higher level of scrutiny. Following the development of the domains, data grouped within each domain were then split into more specific groups. Subdomains captured the richness of the dataset and enabled greater distinction between complaints within each domain grouping. This step was again completed via consensus of the research team. The dataset and domain groupings were analyzed using a more interpretative level of scrutiny, rather than simply relying on the linguistic terms of the dataset alone. This stage involved paying meticulous attention to the corresponding examples and quotes for each domain in order to interpret the underlying concepts and semantics intended by the original investigator. The researchers ensured that the data grouped within each subdomain were representative of the subdomain. This required the researchers to move back and forth between the data to ensure the data were placed appropriately within the subdomains and to identify any overlap or differences in the emerging domain and subdomain groupings. Again, subdomain labels were created using representative words or phrases from within the dataset, thus adopting a bottom-up approach. At this point, it was observed that some of the subdomain items combined several aspects of complaint and could therefore potentially be allocated to more than one subdomain. This was particularly true for examples or quotes that stated an experience with an underlying emotional construct; for example, “frustration in communicating at work” or “upset to know others are aware of hearing problem.” In these cases, the individual complaint or example were assigned to multiple subdomains. There were some domains and subdomains that emerged from multiple data items. However, the development of a subdomain was not based on the number of data items reporting that particular subdomain, but based on the uniqueness of the construct contained within the data. For example, a subdomain could have emerged from data contained within one item if that item could not be consolidated to another subdomain.

#### Expressing the synthesis

We collated the domain groupings from the two perspectives to create two frameworks: one for the PHI and one for the communication partner. These domains sit within a very broad scope of hearing loss complaints, ranging from listening-related complaints to emotional consequences. As described earlier, this breadth guided our decision to structure the frameworks in a hierarchical manner with supra-domains, domains, and subdomains.

## Results

### Study Selection

**Figure 1.**
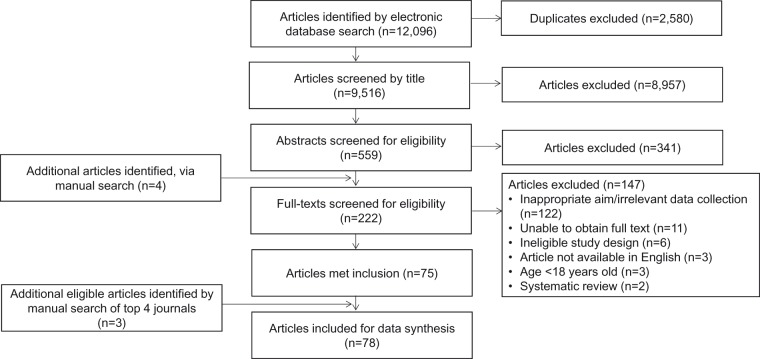
PRISMA flow diagram of study publications. PRISMA = Preferred Reporting Items for Systematic reviews and Meta-analyses.

The electronic search identified 12,096 studies in total: Cos Conference Papers Index (*n*=231), Cumulative Index to Nursing and Allied Health Literature (; *n*=753), Excerpta Medica Database (*n*=4,484), PubMed (*n*=1,697), Web of Science (*n*=4,906), and Google Scholar (*n*=25). The flow of studies through the review process is illustrated in Figure 1. There were 2,579 duplicates, leaving 9,516 studies for title screening. Title screening (VV) removed a further 8,957 studies leaving 559 studies for abstract screening (VV and DAH). At this stage, any record judged as potentially relevant by either author or any record with no abstract was taken forward to full-text reading. Abstract screening removed 341 studies. In total, 222 studies were eligible for full-text review, with this number including four studies identified by the manual search of reference lists. Eight studies subsequently had to be excluded as the research team were unable to obtain a full text, and two were excluded because they were not available in English (Badran, 2001; Sebastian, Varghese, & Gowri, 2015). VV and DAH independently reviewed the remaining 214 full texts against the inclusion criteria. A total of 75 studies met inclusion at this point. A further three eligible studies were identified following the manual search update. For these additional studies, the title and abstracts were screened by two researchers. Therefore, a total of 78 studies met inclusion. All reasons for exclusion were agreed between VV and DAH and are reported in Figure 1. Full citations of these 78 included articles can be found in the Supplementary file (see Supplementary file C).

### Study Characteristics

Most of the included studies focused on the impact of hearing loss on the PHI only (*n*=49). Fewer studies investigated its effects on the communication partner (*n*=11), but there were another 18 publications exploring both perspectives as part of the same study. The age of participants ranged from 18 to 92. Sixty-four studies reflected the views of both men and women; two publications recruited men only (Hetu, Riverin, Getty, Lalande, & St-Cyr, 1990; Jonsson & Hedelin, 2012), one recruited women only (Magilvy, 1985), and 10 studies did not report gender. Sample sizes ranged from *n*=9 (Jonsson & Hedelin, 2012) to *n*=4,266 (Stephens, Lewis, Charny, Farrow, & Francis, 1990). In terms of data collection methods, 44 studies used questionnaires and 34 used qualitative methods. Of those 34 studies, only 11 reported the questions used to elicit complaints, and of those only 9 used open-worded questions: six with people with hearing loss (Hetu, Riverin, Lalande, Getty, & St-Cyr, 1988; Jonsson & Hedelin, 2008; Kelly & Atcherson, 2011; Stephens et al., 1990; Wallhagen & Stawbridge, 2009; Yorgason, Piercy, & Piercy, 2007) and three with communication partners (Lormore & Stephens, 1994; Yorgason et al., 2007); Stephens, France, & Lormore, 1995). Across all included studies (quantitative and qualitative studies), a total number of 996 complaints were extracted and analyzed (622 patient-reported complaints and 374 communication partner-reported complaints). These extracted problems came from studies that investigated both the auditory as well as the nonauditory day-to-day implications of hearing loss.

### Synthesis of Extracted Data

The primary objective of this review was to collect and synthesize the complaints with hearing loss for people with hearing loss- and communication partner-reported complaints of hearing loss. In the following section, the main findings are presented separately for the two domain grouping perspectives (people with hearing loss and communication partner). Using the domain groupings, two frameworks were developed, termed *Domains of hearing loss-Person with hearing loss* (DoHL-P) and *Domains of hearing loss – Communication partner* (DoHL-CP). All communication partner participants in studies that investigated this perspective were spouses or partners of the PHI. Included studies typically reported a mixture of domains and illustrative examples, either in terms of individual questionnaire items or participant quotes. For this reason, we considered the data items supported in the primary data synthesis to be equivalent, irrespective of the study design, or analysis methodology. The four additional studies identified in the updated manual search were subjected to the same data collection. The extracted complaints of hearing loss were considered with our frameworks to decipher if any new information or complaints were reported in these studies. However, these studies did not identify any new domains or subdomains that were not already in our frameworks.

Our classification scheme comprises of three overarching supra-domains. “Auditory” refers to domains relating to perception of sound and speech. “Social” refers to domains that represent the impact of hearing loss on activities with friends and family, as well as attitudes to hearing loss. “Self” refers to domains relating to self-perception and personality. Each supra-domain encapsulated constituent domains and subdomains. “Auditory” had 4 domains and 35 subdomains (Table 1). “Social” had 5 domains and 18 subdomains (Table 2) and “Self” had 5 domains and 28 subdomains (Table 3). Many of the domain-level appear in both frameworks, and this was true for the subdomain complaints associated with communicating (“Auditory,” Table 1) and the perceived role of the communication partner (“Self,” Table 3), but not for other subdomains. The tables list all the domains, and in the following text, we highlight some. The domains reported by patients only will be reported first, followed by those domains reported by communication partners only, and then the domains reported by both. The “Auditory” supra-domain contained the highest number of subdomains. In total, we found 58 subdomains for the PHI and 37 for the communication partner. Each domain will be described in turn with the number of extracted data items used to form each domain denoted by *n*. Note that the number of complaints only marks how often the complaint appears in the surveyed literature; it does not determine the importance of a complaint, either to an individual or everyone.

**Table 1.** List of Identified Domains and Subdomains That Were Classified Within the “Auditory” Supra-Domain.

Domain	DoHL-P		Both	DoHL-CP
Hearing				
	• Hearing warning sounds • Hearing telephone ring • Hearing the doorbell • Hearing television and radio sounds	• Hearing distant sounds • Hearing environmental sounds • Hearing speech sounds • Sound Localization		
Listening				
	• Listening to speech on the telephone • Listening to speech in noisy environments • Listening to birdsong • Listening to music • Pleasure of listening to music • Listening to quiet speech (whispering)	• Listening to speech (at work) • Listening to speech (speech in general) • Listening to speech in a group of talkers • Listening to speech in a travelling vehicle • Listening to speech in public places • Listening to speech in quiet environments	• Raising the volume of the television/radio	
Communicating				
	• Asking people to repeat • Communicating at work • Communicating, general conversation	• Communicating, speech in quiet • Conversing on the telephone • Reduced communication	• Conversing in a group of talkers • Conversing in noisy environments • Conversing one to one • Having to speak on behalf of partner with hearing loss • Reduced spontaneous conversation	• Having to repeat
Speaking				
			• Speaking with a loud voice	

*Note.*Column 2 displays the subdomains that appear in Domains of Hearing Loss-Persons with hearing loss (DoHL-P) only. Column 3 shows the subdomains that appear in both frameworks. Column 4 displays the subdomains that appear in Domains of Hearing Loss-Communication partners (DoHL-CP) only.

**Table 2.** List of Identified Domains and Subdomains That Were Classified Within the “Social” Supra-Domain.

Domain	DoHL-P	Both	DoHL-CP
Interventions			
	• Expectations of rehabilitation • Technical problems with hearing aid		
Social life			
	• Social withdrawal • Altered social interactions		• Goes out alone • Reduced enjoyment of social activities
Isolation			
	• Sense of isolation		• Don’t go out much as a couple • Isolated as a couple at social events
Occupational			
	• Abilities to perform duties at work • Opportunities at work		
Relationships			
	• Relationship with family members	• Relationship with spouse	• Effects on intimate relationship • Misunderstandings • Relationship conflicts • Threat to relationship

*Note.* Column 2 displays the subdomains that appear in Domains of Hearing Loss-Persons with hearing loss (DoHL-P) only. Column 3 shows the subdomains that appear in both frameworks. Column 4 displays the subdomains that appear in Domains of Hearing Loss-Communication partners (DoHL-CP) only.

**Table 3.** List of Identified Domains and Subdomains That Were Classified Within the “Self” Supra-Domain.

Domain	DoHL-P	Both	DoHL-CP
Role of CP			
			• Having to answer the telephone • Having to say that phone is ringing • Accommodating to the hearing loss • Having to act as an interpreter • Speech production by partner
Emotions			
	• Embarrassment • Rejection • Worry	• Frustration • Anger • Upset	• Burden of adjustment • Having to raise the TV/radio volume • Emotional consequences on relationship • Guilt • Stress
Effort and fatigue			
	• Listening effort	• Feelings of fatigue	• Effort and fatigue of having to accommodate • Effort involved in communication • Effort of having to act as an interpreter • Effort of having to repeat
Identity			
	• Feelings of inadequacy/self-esteem	• Self-image	
Stigma			
	• Stigma of hearing aids • Pretending to understand speech	• Denial • Stigma of hearing loss	

*Note.* Column 2 displays the subdomains that appear in Domains of Hearing Loss-Persons with hearing loss (DoHL-P) only. Column 3 shows the subdomains that appear in both frameworks. Column 4 displays the subdomains that appear in Domains of Hearing Loss-Communication partners (DoHL-CP) only.

### Primary Objectives: Patient-Reported Domains

#### Auditory: Hearing

This domain corresponds to the passive function of hearing, the ability to access sound, but not always in the context of listening to speech. The seven subdomains within this domain describe difficulties in hearing various types of sounds. The most commonly reported complaint was hearing warning sounds (*n*=8 complaints) such as fire alarms. Specific problematic situations reported include hearing the telephone ring (*n*=7 complaints), television/radio sounds (*n*=5 complaints), and the doorbell (*n*=4 complaints). While these complaints were not directly reported in the context of hearing in the DoHL-CP, communication partners did observe the increase in the volume of the television and constantly having to answer the telephone.

#### Auditory: Listening

For people with hearing loss, the most commonly reported problem of the 12 subdomains was listening to speech in noisy environments (*n*=17 complaints).

#### Social: Interventions

This domain focuses primarily on the issues associated with any interventions an individual receives for their hearing loss. Its two subdomains cover the limitations of some interventions and unrealistic expectations of hearing aids (*n*=3 complaints) “ … a hearing aid would be wonderful if they could solve the problem … “ (Claesen & Pryce, 2012, p. 282).

#### Social: Occupational impact

This domain refers to problems experienced in hearing-impaired person’s place of work, as a result of their hearing difficulties. The three subdomains describe the impact of hearing loss on everyday work-life, and particularly in relation to potential implications of their hearing loss on job security (*n*=4 complaints); for example, “Originally I was told that I would lose my job if I needed to use interpreters” (Punch, Hyde, & Power, 2007, p. 511).

### Primary Objectives: Communication Partner-Reported Domains

#### Self: Role of Communication Partner

This domain refers to any additional responsibilities or roles the communication partner has had to take on as a result of the limitations hearing loss has imposed on their communication partner that prevents them from carrying out certain tasks. The five subdomains within this domain describe roles relating to listening and communication in the home and social settings. The most commonly reported task reported by communication partners was having to answer the telephone (*n*=7 complaints) as well as having to tell the PHI the phone is ringing (*n*=2 complaints):

having to answer the telephone seemed to be that’s a source of annoyance because it’s never for me … and I have to take the call and then I have to go through the Oh yeah, I’m well thanks, How are you? (Scarinci, Worrall, & Hickson, 2009a, p. 2092)

This is due to the complaints relating to the telephone in the patient domains, whereby a PHI avoided having to answer the phone due to difficulty hearing the telephone ring, as well as listening to conversation while on the phone. Another reported subdomain, having to act as an interpreter (*n*=3 complaints), refers to communication partners having to speak on behalf of the PHI, particularly in social situations, “ … by the time you’ve tried telling him what they have said, they’ve moved on … “ (Morgan-Jones, 1998, p. 68). Communication strategies or tactics employed by the partner to aid communication with the person with the hearing loss were also explicitly reported (*n*=2 complaints) which more generally captures the accommodations by communication partners to the PHI. One explicitly reported strategy, “speech production by partner” (*n*=4 complaints), represents the communication partner having to raise their voice in order to facilitate effective communication with the PHI.

Most of the difficulties encountered by the PHI were also noticed by the partner, and vice versa. For example, communication partners acknowledged that the PHI does not often hear the telephone and PHI acknowledged that they were more reliant on the communication partner to answer the telephone. Partners acknowledged the PHI withdrawal from social situations as well as their own compensation to engage on behalf of the PHI in response to the withdrawal.

### Primary Objectives: Person With Hearing Loss- and Communication Partner-Reported Domains

#### Auditory: Listening

This domain refers to difficulties experienced when hearing is purposefully engaged, implying some degree of attentional effort, especially but not always in the context of listening to speech. The 13 subdomains (see Table 2) within this domain describe listening problems experienced in everyday situations both in the home environment and in public spaces. The subdomain “raising the volume of the television/radio” appears in both frameworks. Another common subdomain was listening to speech on the telephone (*n*=10 complaints) to the extent that several examples reported individuals with hearing loss avoiding taking phone calls (Hetu, Jones, & Getty, 1993; Hetu et al., 1990; 1988; Miyakita, Ueda, Zusho, & Kudoh, 2002). The corollary of that was observed in one partner domain, “Role of the communication partner,” where taking responsibility for answering the telephone was very common (*n*=7 complaints) across the 29 studies that questioned partners. The next frequently reported difficulty for people with hearing loss was listening to speech in noisy environments (*n*=17 complaints). Listening to the television and radio was also common, resulting in having to raise the volume (*n*=14 complaints). There was a direct equivalent for communication partners, which was that of having to listen to the television or radio louder than what they would normally prefer (*n*= 9 complaints).

#### Auditory: Communicating

This domain refers to difficulties experienced when actively participating in conversation, where there is a mutual exchange of spoken information between at least two people. All of the 12 subdomains describe problems experienced in conversational settings and these can be in the home as well as in public places. For people with hearing loss, the most commonly reported problems were participating in general conversation and in conversation with a group of talkers (*n*=13 and *n*=12 complaints, respectively). Next concerned asking people to repeat things (*n*=11 complaints). The result of that for communication partners was having to repeat to their partner which was mentioned seven times (*n*=7 complaints). The second most frequently reported challenge for partners was conversing on a one-to-one basis (*n*=5 complaints). Examples and quotes indicate that this subdomain predominantly refers to conversations with the hearing impaired spouse: “When you’ve spent forty years able to converse easily and then one goes deaf it’s very difficult to adjust … “ (Morgan-Jones, 1998, p. 65); “my husband sometimes gets annoyed because I can’t hear and he has to keep repeating” (Hass-Slavin, McColl, & Pickett, 2005, p. 331).

#### Auditory: Speaking

This domain refers to changes in the volume of one’s speaking voice that can occur as a result of hearing loss. There was just one subdomain, “speaking with a loud voice,” that appears in both frameworks. For people with hearing loss, this related to increases in the volume in their voice since the onset of hearing loss (*n*=2 complaints). For communication partners, speaking problems also related to an increase in volume of their own voice when talking to their hearing-impaired partner (*n*=3 complaints). For example, “I have to raise my voice” (Govender, Maistry, Soomar, & Paken, 2014, p. 52).

#### Social: Social life

Social withdrawal was the most frequently reported subdomain overall (*n*=42 complaints). There was no overlap in the four subdomains across the DoHL-P and DoHL-CP. Quotes given by people with hearing loss made reference to the inability to fully engage in a social event or social gathering, and physically removing themselves from the situation due to experienced difficulties. For example, “I find myself avoiding company because conversation is too much effort” (Hallam & Brooks, 1996, p. 205) and “ … there was a party I end up in the kitchen because it is quiet there. If two or three are talking I can’t hear. Then I left” (Wanstrom et al., 2014, p. 32). There were 14 mentions of the changes and quality of social interaction, for example, “I do communicate socially but I find, I suppose because I am deaf, I don’t like conversations to be so long … that one has to think ‘Now what exactly is that person saying?’” (Morgan-Jones, 1998, p. 78). Social life was the domain with the highest reported data items across the literature included in our review.

Communication partners made similar complaints. Reduced enjoyment of social activities due to their partner’s hearing loss was most common (*n*=7 complaints). For example, “ … he’s not participating in the actual conversation and there’s just all this noise going on around him he just switches off” (Scarinci, Worrall, & Hickson, 2009b, p. 146). Indeed, one communication partner complained that such difficulties had resulted in him or her attending social events alone, “He might accept a social invitation initially but he could also pull out” (Scarinci et al., 2009b, p. 146).

#### Social: Isolation

The domain “sense of isolation” encompassed feelings of separation and exclusion from others, especially in relationships or during social gatherings. Again, there was no overlap in the three subdomains across frameworks. For people with hearing loss, the perception of isolation was very much in the context of themselves in certain social situations (*n*=26 complaints). For example, “My hearing loss makes me feel isolated from other people” (Hallam & Brooks, 1996, p. 205) and “I feel a bit left-out” (Morgan-Jones, 2001, p. 45). For communication partners, complaints referred to their general sense of being part of a couple (*n*=8 complaints). For example, “We don’t go along to our senior’s group anymore … isolated at parties” (Scarinci et al., 2009b, p. 2092). Furthermore, they reported feeling isolated as a couple at social events (*n*=3 complaints).

#### Social: Relationships

People with hearing loss and communication partners both acknowledged that hearing loss can have negative effects on personal relationships. Of the six submains, the subdomain “relationship with spouse/partner” appears within both frameworks. For people with hearing loss, changes in their relationship with family members and their spouse or partner were often attributed to their hearing loss (*n*=8 and *n*=5, respectively). Spouses or partners were often identified as the primary motivator for seeking an audiological appointment usually as a result of the strain on communication: “My wife threatened me with divorce” (Claesen & Pryce, 2012, p. 283). People with hearing loss also attributed communication breakdown in their relationship to their hearing difficulties: “acknowledge responsibility in communication breakdown” (Claesen & Pryce, 2012, p. 283).

Negative effects on relationships were more prominent with communication partners than people with hearing loss. In particular, effects on the intimate aspects of the relationship were frequent (*n*=8 complaints). For example, “My partner’s hearing difficulties has an effect on our intimate relationship” (Govender et al., 2014, p. 53), “I withdraw from my partner and we do things alone” (Govender et al., 2014, p. 53).

Hearing loss sometimes also results in greater conflict, threat, and misunderstanding in relationships (*n*=3, *n*=2, and *n*=1, respectively). For example, “there is no use in discussing the problem with him…it does not work, it always ends up in a conflict” (Hallberg, 1999, p. 53) and “I’ve threatened to leave him to fend for himself it he didn’t toe the line” (Scarinci et al., 2009a, p. 2092).

#### Self: Emotions

Several emotional responses to hearing loss, and to the secondary problems caused by hearing loss were reported across both frameworks (Table 3). In total, there were 11 subdomains. There were a higher number of emotional domains reported by the communication partner literature. Within the communication partner domains, feelings of frustration at their partner for having hearing loss was the highest-reported emotional subdomain. For example, “I understand she’s got a problem but it doesn’t stop me from getting frustrated as hell sometimes” (Scarinci et al., 2009a, p. 2092). For those with hearing loss, negative emotional domains were typically in response to limitations imposed by their hearing loss (*n*=11 complaints); “just can’t hear what they’re saying to me … it’s just awful” (Claesen & Pryce, 2012, p. 279). The most common emotion subdomain in the partner domains was frustration (*n*=14 complaints) of the difficulties hearing loss imposed on several aspects of life such as at the compensation for the social dependence of the impaired spouse or having to undertake additional responsibilities. Another reported subdomain was the burden of adjustment to hearing loss (*n*=4 complaints) experienced by communication partners: for example, “I feel that it’s actually the other people who are with him who suffer more than him because I think they’ve got to adapt their living style rather than him” (Scarinci et al., 2009a, p. 2092). The emotional consequences of having to raise the volume of the television or radio was frequently reported (*n*=6 complaints); for example, “What I find is when it gets up too high, it aggravates me. I don’t get any pleasure out of it … I’m not having a happy time” (Scarinci et al., 2009a, p. 2092). This corresponds to the earlier result that in the Auditory: Listening domain, loudness of the television or radio was frequently reported as a problem.

#### Self: Effort and fatigue

This domain refers to the additional resources required to listen and participate in conversation. It has six subdomains. The subdomain “feelings of fatigue” appears in both frameworks (patient: *n*=13 complaints; partners: *n*=12 complaints) and across both frameworks. People with hearing loss frequently reported exerting greater effort in order to listen and follow a conversation (*n*=8 complaints), and consequently reported feelings of fatigue (*n*=6 complaints). For example, “I fell asleep when I was at the meeting and after they said to me I know how you fell asleep, because you couldn’t concentrate the whole period” (Granberg, Pronk, et al., 2014, p. 783). For communication partners, complaints related to effort involved in communication comprised the highest-reported subdomain, particularly due to frequent misunderstandings and communication breakdowns.

#### Self: Identity

This domain refers to the way hearing loss has negatively changed an individual’s perception of themselves, or, in the case of communication partners, their perception of themselves as a couple. There are just two subdomains. The subdomain “self-image” appears in both frameworks. The subdomain feelings of inadequacy or self-esteem was highly reported (*n*=17 complaints): for example, “ … there’s no connection, you can’t hear. Well, it actually becomes part of your self-esteem as well” (Jonsson & Hedelin, 2012, p. 318). Persons with hearing loss complaining of feeling bothersome to others, “I don’t find it too much of a problem but other people do,” particularly during conversation while having to ask people to repeat themselves. Another frequently reported complaint was feelings in relation to negative self-image (*n*=6 complaints); “You feel incomplete … mutilated” (Jonsson & Hedelin, 2012, p. 318). For communication partners, four complaints regarding image related to striving to maintain the social image of themselves and the PHI as a couple.

#### Self: Stigma

In this domain, the four subdomains reflect the personal stigma that is associated with hearing loss or hearing aids, and the behaviors resulting from those societal beliefs, as opposed to society in general. The subdomains “denial” and “stigma of hearing loss” appears across both frameworks. People with hearing loss particularly identified complaints of stigma either of hearing loss (*n*=9 complaints) or hearing aids (*n*=7 complaints). For example, “having been diagnosed, I feel I have labelled myself” (Morgan-Jones, 2001, p. 88) and stigma of hearing aids, “I think that if you wear a hearing aid, people tend to ignore you” (Hallam & Brooks, 1996, p. 206). Admitting denial was a recurring complaint (*n*=11 complaints). For example, “When I became conscious of it the hearing loss I kept trying to deny [it]” (Yorgason et al., 2007, p. 219). Wanting to conceal hearing loss or minimize the effort of participating in conversation was also frequently reported (*n*=7 complaints). For example, “Lots of times it is useful if you tell somebody something and they say, ‘OK.’ Rather than no response” (Yorgason, Piercy, & Piercy, 2007, p. 221).

Communication partners mentioned only the stigma of hearing loss (*n*=6 complaints). In particular, this was made in reference to aging or a sign of “getting old” (see also the Self: Identity subdomain).

Communication partners also reported being in denial (*n*=3 complaints) or unwilling to accept their partner’s hearing difficulties were due to hearing loss “ … as a spouse you actually perpetuate. I suppose you deny it yourself as a well as a spouse. You say ‘Oh well, maybe it isn’t as bad as that, maybe I’m just impatient … ‘“ (Scarinci et al., 2009b, p. 147).

### Secondary Objectives: Comparison to Brief ICF Core Set for Hearing Loss

The first of the secondary objectives compared DoHL-P with the brief ICF Core Set for hearing loss. This comparator is of interest because it has integrated patient and professional perspectives to create a comprehensive list of categories relevant to adults’ hearing loss (Danermark et al., 2013) and it was developed to provide an assessment of an individual’s functioning. The emphasis on the patient themselves means that the comparison to our frameworks can highlight what is missed by not including their communication partner.

We conceptually mapped the domains in our DoHL-P to the 27 domains of the brief ICF Core Set using the descriptive labels of each domain and the ICF definitions to assist in interpretation where differences were simply due to terminology (e.g., ‘occupational impacts’ versus ‘remunerative employment’; Table 4).

**Table 4.** Table Showing How We Have Mapped Our Findings Onto the Existing Domain Framework Defined by the Brief ICF Core Set for Hearing Loss.

DoHL-P	DoHL-P and ICF brief core set	Unique to ICF brief core set for hearing loss
Isolation Social Life Effort and Fatigue Identity Stigma Role of Communication Partner	**Hearing** (Hearing functions b230, sound e460, Sensations associated with hearing aid/vestibular b240) **Emotions** (Emotional functions b152) **Communicating** (Communicating with receiving spoken messages d310) **Relationships** (Immediate family e310, family relationships d760, Individual attitudes of immediate family members e410)) **Occupational** (Remunerative employment d850) **Speaking** (Conversation d350) **Listening** (Listening d115) **Interventions** (Using communication devices and techniques d360)	Temperament and personality functions (b126) Functions (attention, memory, seeing) (b140, b144, b210) Structure of (brain, external ear, middle ear, inner) (s110, s240, s250, s260) Handling stress and other psychological (d240) School education (d820) Community life (d910) Products and technology (e125) Societal attitudes (e460) Health services, systems, and policies (e580) Health professionals (e355)

*Note.*We judged some of the ICF domains not to be within the scope of our systematic review question. These were as follows: s110; s240; s250; s260; b126; b140; b144; b210; d240; d820; e125; e355, e580.

Eight of our domains clearly mapped onto those of the brief ICF Core Set for hearing loss, namely “Auditory: Hearing,” “Self: Emotions,” “Auditory: Communicating,” “Social: Relationships,” “Social: Occupational,” “Auditory: Speaking,” “Auditory: Listening,” and “Social: Interventions”. The remaining six domains appeared to be unique to DoHL-P: “Social: isolation,” “Social: social life,” “Self: effort and fatigue,” “Self: stigma,” “Self: identity,” and “Self: role of communication partner.” This result shows that all the Auditory domains and some of the Social domains are included in the ICF, but only one of the Self domains.

With respect to “Self: identity,” the brief ICF Core Set does contain the domain “societal attitudes” that is defined as “ … beliefs held by a social group about other individuals … “ but this represents a different concept than the one identified in our review which was concerned specifically with the way hearing loss negatively changed the person’s perception of themselves. Overall, some of the patient-reported implications are *not* represented within the brief ICF Core Set for hearing loss. Furthermore, while the ICF framework was designed to be widely disseminated across the field of audiology (Granberg, Dahlstrom, et al., 2014; Granberg, Pronk, et al., 2014), it exclusively focuses on the person with the health condition and excludes aspects relating to the communication partner. If the ICF framework solely was used to guide clinical practice (such as by framing the patient assessment appointment), then it would risk diminishing the value of implementing family-centred care principles in audiological practice.

In subsequent analyses, we obtained from the original authors (Granberg, personal communication, December 16, 2015) their data that was coded as “component not covered” within the ICF (Granberg, Pronk, et al., 2014), then mapped those onto the domains and subdomains in the DoHL-P and DoHL-CP using descriptions of each category (Supplementary file D). Due to the generalizability of the category names, the meaning of some of the categories was ambiguous and could not be mapped onto the framework such as “mutations” and “waking up,” and therefore was placed in the “miscellaneous” category. In other cases, some categories were interpreted as being applicable to more than one domain such as “participation” and “behaviour of others.” It is of interest that all of the “components-not covered” that were mapped onto the communication partner domains fell into the subdomain “Self: role of communication partner.” This may provide a reason as to why these could not be coded within the ICF since the ICF only captures problems experienced by the PHI. The absence of these categories from the ICF potentially excludes information regarding the broader impact of hearing difficulties and relationship changes due to hearing loss that are considered important to people with hearing loss. Issues concerning stigma and identity are not routinely considered by questionnaires such as the HHIE (Ventry & Weinstein, 1982); yet, excluding those personal aspects of hearing loss regarded to be important by patients and their partners is not in line with patient-centred communication principles and may have negative consequences for audiological assessment and management decisions (Ekberg, Grenness, & Hickson, 2014; Grenness, Hickson, Laplante-Levesque, Meyer, & Davidson, 2015).

### Secondary Objectives: Severity of Hearing Loss

We also investigated the pattern of reported complaints in relation to severity of hearing loss, where hearing status was adequately specified. Only 27 studies reported the hearing status of participants, and even in these studies, there was considerable variation in the way hearing loss was reported (e.g., mean pure-tone audiometric hearing thresholds, sensorineural or conductive, or self-reported judgment). Where possible, we graded severity into three categories using UK audiometric descriptors (British Society of Audiology, 2011) based on the average of the pure tone hearing threshold levels at 250, 500, 1000, 2000, and 4000 Hz: (a) mild hearing loss (20–40 dB HL), moderate hearing loss (41–70 dB HL), and severe to profound hearing loss (≥71 dB HL). From these, a subset of 18 studies could be classified (7 mild; 8 moderate; 2 severe-to-profound). Extracted domains and complaints for mild and moderate hearing losses were split according to these categories. We found that in total, there were 70 individual complaints reported from studies that stated hearing loss severity. Of those, 17 complaints were reported from studies using participants with mild hearing loss, 44 complaints from studies with moderate hearing loss, and 9 complaints from studies exploring profound hearing loss. The breakdown of hearing loss-associated complaints relating to hearing loss severity can be found in Supplemental file E. There was insufficient data to categorize according to the extracted subdomains for the severe-to-profound hearing loss severity.

Complaints relating to communication and speech comprehension were the most commonly reported across the included studies, as well as emotional subdomains. The data extracted in the present review showed considerable overlap across hearing loss severity and the domains extracted. The social impact of hearing loss, particularly social withdrawal was also represented across both (mild-moderate) hearing loss severities. The classification of hearing loss severity, however, was based on pure-tone averages only. Information regarding the onset of hearing loss and duration of hearing aid use are both important pieces of information for examining the lived experiences of hearing loss as a function of the time that an individual has to adapt to their hearing loss. Neither of these parameters was consistently reported.

### Quality Assessment

Since we used an entirely data-driven approach, subdomains can *only* enter our frameworks if they have been mentioned by a patient or communication partner and then reported in a paper. That is, should a potential topic not be covered, or a patient forgets to say something, then potentially important data could be missed. In general, this cannot be ruled out, and so some measure of its potential extent can be gauged by formally assessing the quality of the studies. Such appraisals give a general overview of the quality of reporting of the included studies and is considered an important component of reviews (Centre for Reviews and Dissemination, 2009).

The application of quality criteria to qualitative research is widely debated (Dixon-Woods, Shaw, Agarwal, & Smith, 2004). This is due to the lack of distinction between the quality and process of a study, and those concerned with transparency of reporting (Dixon-Woods et al., 2004). Several quality assessments for qualitative research have been developed. However, there is a lack of consensus as to those that should be routinely adopted. This is also due to the argument that different qualitative methods need to be appraised in different ways (Centre for Reviews and Dissemination, 2009), and appraising the most important qualities of qualitative studies can be challenging (Dixon-Woods et al., 2004).

We chose the Critical Appraisal Skills Programme (CASP, 2016) to determine the assessment (Vas et al., 2016). This is a 10-item quality appraisal tool developed explicitly for use in systematic reviews (Centre for Reviews and Dissemination, 2009). The CASP checklist was applied to the 34 qualitative studies included in the present review. Two researchers (VV and DAH) independently appraised the qualitative studies using the CASP checklist given in Table 5. Where there was disagreement in appraisal for a particular study, this was resolved through discussion. Overall, reporting of the studies was adequately detailed and relevant considerations undertaken. Where studies scored lower on the CASP checklist was in relation to justifying the methods used in the study; 24 out of the 34 qualitative studies. Another item that did not score as well was a description of risk of bias of the researcher conducting the qualitative research (how data collection might have been affected by the investigator-participant relationship), with 20 studies reporting this.

**Table 5.** Quality Appraisal of Qualitative Studies Using CASP Checklist.

	Study Aims clearly stated	Appropriate methods	Justification of methods	Recruitment strategy given	Data collection described	Researcher bias discussed	Study ethics description	Description of data analysis	Description of results	Value of research
Magilvy (1985)	Yes	Yes	No	Yes	Yes	No	Can’t tell	No	Yes	Yes
Thiede (1986)	Yes	Yes	Yes	Yes	Yes	Can’t tell	Yes	Yes	Yes	Yes
Hetu et al. (1988)	Yes	Yes	Yes	Yes	Yes	Yes	Yes	Yes	Yes	Yes
Hetu et al. (1990) (Study 1)	Yes	Yes	Can’t tell	Yes	Yes	Yes	Yes	Yes	Yes	Yes
Hetu et al. (1990) (Study 2)	Yes	Yes	Yes	Yes	Yes	Yes	No	No	Can’t tell	Yes
Bade (1991)	Yes	Yes	Yes	Yes	Can’t tell	Yes	Yes	Yes	Yes	Yes
Hallberg and Barrenas (1993)	Yes	Yes	Yes	Yes	Yes	Yes	Yes	Yes	Yes	Yes
Hetu et al. (1994)	Yes	Yes	No	Yes	Yes	Yes	Yes	Yes	Yes	Yes
Cowie, Watson, Kerr, and Douglas- Cowie (1995)	Yes	Yes	No	Can’t tell	Yes	Yes	Yes	Yes	Yes	Yes
Stephens et al. (1995)	Yes	Yes	Can’t tell	No	Yes	Can’t tell	Yes	Yes	Yes	Yes
Tesch-Romer (1997)	Yes	Yes	Yes	Yes	Yes	Yes	Yes	Yes	Yes	Yes
Hallberg (1999)	Yes	Yes	Yes	Yes	Yes	Yes	Yes	Yes	Yes	Yes
Gething (2000)	Yes	Yes	Can’t tell	Yes	Can’t tell	Can’t tell	Yes	Yes	Yes	Yes
Laroche, Garcia, and Barrette (2000)	Yes	Yes	No	Can’t tell	Yes	Yes	Can’t tell	Yes	Yes	Yes
Brooks, Hallam, and Mellor (2001)	Yes	Yes	Yes	Yes	Yes	Yes	Yes	Can’t tell	Yes	Yes
Morgan-Jones (2001)	Yes	Yes	No	Can’t tell	Can’t tell	No	No	Yes	Yes	Yes
Hass-Slavin et al. (2005)	Yes	Yes	Can’t tell	Yes	Yes	Yes	No	No	Yes	Yes
Ross and Lyon (2007)	Yes	Yes	Yes	Yes	Yes	Yes	Can’t tell	Yes	Yes	Yes
Punch et al. (2007)	Yes	Yes	Yes	Yes	Yes	Yes	Yes	Yes	Yes	Yes
Yorgason et al. (2007)	Yes	Yes	Yes	Yes	Yes	Yes	Yes	Yes	Yes	Yes
Hallam, Ashton, Sherbourne, and Gailey (2008)	Yes	Yes	Yes	Yes	Yes	Can’t tell	Yes	Yes	Yes	Yes
Hidalgo et al. (2008)	Yes	Yes	Yes	Yes	Yes	Yes	Yes	Yes	Yes	Yes
Jennings and Shawb (2008)	Yes	Yes	No	Can’t tell	Can’t tell	No	No	Can’t tell	Yes	Yes
Scarinci, Worrall, and Hickson (2009a)	Yes	Yes	Yes	Yes	Yes	Yes	No	Yes	Yes	Yes
Wallhagen (2010)	Yes	Yes	Yes	Yes	Yes	Can’t tell	Yes	Yes	Yes	Yes
Kelly and Atcherson (2011)	Yes	Yes	Yes	Yes	Yes	Yes	Can’t tell	Yes	Yes	Yes
Claesen and Pryce (2012)	Yes	Yes	Yes	Yes	Yes	Can’t tell	Yes	Yes	Yes	Yes
Jonsson and Hedelin (2012)	Yes	Yes	Yes	Yes	Yes	Can’t tell	Yes	Yes	Yes	Yes
Manchaiah and Stephens (2013)	Yes	Yes	Yes	Yes	Yes	Yes	Yes	Yes	Yes	Yes
Ekberg et al. (2014)	Yes	Yes	Yes	Yes	Yes	Yes	Yes	Yes	Yes	Yes
Granberg, Pronk, et al. (2014)	Yes	Yes	Yes	Yes	Yes	Can’t tell	Yes	Yes	Yes	Yes
Preminger and Laplante- Levesque (2014)	Yes	Yes	Yes	Yes	Yes	No	Yes	Yes	Yes	Yes
Wanstrom et al. (2014)	Yes	Yes	Yes	Yes	Yes	Yes	Yes	Yes	Yes	Yes
Heffernan et al. (2016)	Yes	Yes	Yes	Yes	Yes	Yes	Yes	Yes	Yes	Yes

*Note.* CASP = Critical Appraisal Skills Programme.

The remaining 44 studies (all quantitative) were subjected to a second quality appraisal as defined in the protocol (Vas et al., 2016). Studies were assessed for (a) reporting of sample size, (b) reporting a wide variety of ages (mean and SD), (c) reporting of participant’s gender, (d) reporting of inclusion and exclusion criteria, (e) reporting ethical considerations, and (f) reporting of data analysis. Each criterion was scored 0 (“no”), 1 (“can’t tell”), or 2 (“yes”): a score of zero indicated the study did not report the item being assessed, a score of one indicates that a judgment could not be made as to whether the item was taken addressed based on what the authors have included, and a score of two indicates the study reported and addressed the item in question well. Table 6 reports the results. The mean quality score across all papers and criteria was 1.0 with a distribution of 121 “no’s,” 122 “yes’s,” and 39 “not clears.” Across papers, 10 had at least three criteria marked at “no,” whereas only 13 had at least three criteria marked at “yes.” Across criteria, the mean scores were 1.5 for sample size, 0.1 for sample size, 1.5 for age, 1.8 for gender, 0.9 for inclusion or exclusion, 0.6 for ethics, and 1.2 for analysis. We conclude that there is much room for improvement in the reporting of methods, especially in the criteria of sample size and ethics.

**Table 6.** Quality Appraisal of Quantitative Studies.

	Reporting of sample size	Reporting of participants age	Reporting of participants gender	Reporting of inclusion and exclusion criteria	Reporting of ethics	Reporting of data analysis	Mean score per study
Newman and Weinstein (1986)	0.0	2.0	2.0	0.0	0.0	0.0	0.7
Garstecki (1987)	0.0	2.0	2.0	0.0	0.0	0.0	0.7
Hetu, Lalonde, and Getty (1987)	0.0	2.0	2.0	2.0	0.0	2.0	1.3
Vesterager, Salomon, and Jagd (1988)	0.0	1.0	2.0	1.0	0.0	0.0	0.7
Martin, Krall, and O’Neal (1989)	0.0	1.0	2.0	0.0	0.0	0.0	0.5
Mulrow et al. (1990)	0.0	1.0	2.0	1.0	2.0	2.0	1.3
Stephens et al. (1990)	0.0	0.0	2.0	0.0	0.0	0.0	0.3
Knutson and Lansing (1990)	0.0	2.0	2.0	1.0	1.0	0.0	1.0
Mulrow et al., (1990)	0.0	0.0	2.0	1.0	0.0	1.0	0.7
Vesterager and Salomon (1990)	0.0	2.0	2.0	2.0	1.0	0.0	1.2
Plath (1991)	0.0	1.0	2.0	1.0	0.0	0.0	0.7
Slawinski, Hartel, and Kline (1993)	0.0	1.0	2.0	0.0	0.0	1.0	0.7
Lormore and Stephens (1994)	0.0	0.0	2.0	0.0	0.0	0.0	0.3
Hallberg and Barrenas (1994)	0.0	2.0	2.0	2.0	0.0	2.0	1.3
Gilbertson, Fusilier, Murch, and Dancer (1996)	0.0	2.0	2.0	2.0	2.0	2.0	1.7
Hallam and Brooks (1996) (Study 1)	0.0	2.0	2.0	0.0	0.0	1.0	0.8
Hallam and Brooks (1996) (Study 2)	0.0	2.0	2.0	0.0	0.0	0.0	0.7
Hallam and Brooks (1996) (Study 3)	0.0	2.0	0.0	0.0	0.0	0.0	0.3
Caissie and Gibson (1997)	0.0	2.0	2.0	0.0	0.0	1.0	0.8
Newman, Jacobsen, Hug, and Sandridge (1997)	0.0	1.0	1.0	1.0	0.0	1.0	0.7
Gatehouse (1999)	0.0	0.0	2.0	0.0	0.0	1.0	0.5
Strawbridge, Wallhagen, Shema, and Kaplan (2000)	0.0	1.0	2.0	0.0	0.0	2.0	0.8
Kochkin and Rogin (2000)	0.0	1.0	2.0	0.0	0.0	2.0	0.8
Albera et al. (2001)	0.0	1.0	2.0	2.0	0.0	0.0	0.8
Morgan-Jones (2001)	0.0	2.0	1.0	2.0	1.0	2.0	1.3
Tsuruoka et al. (2001)	0.0	2.0	2.0	2.0	0.0	2.0	1.3
Espmark, Rosenhall, Erlandsson, and Steen (2002)	0.0	1.0	2.0	0.0	0.0	2.0	0.8
Miyakita et al. (2002)	0.0	2.0	1.0	0.0	0.0	2.0	0.8
Robinson and Hames (2004)	0.0	1.0	2.0	0.0	0.0	0.0	0.5
Stark and Hickson (2004)	0.0	2.0	2.0	2.0	1.0	0.0	1.2
Anderson and Noble (2005)	0.0	2.0	2.0	0.0	0.0	1.0	0.8
Vuorialho, Karinen, and Sorrit (2006)	0.0	2.0	2.0	2.0	2.0	2.0	1.7
Saunders and Forsline (2006)	0.0	2.0	1.0	0.0	0.0	0.0	0.5
Leposavić, Leposavić, Jasović-Gasić, Milovanović, and Nikolić-Balkoski (2006)	0.0	1.0	2.0	0.0	0.0	2.0	0.8
Cox, Alexander, and Gray (2007)	0.0	2.0	2.0	2.0	0.0	0.0	1.0
Helvik et al. (2006)	0.0	2.0	2.0	0.0	1.0	2.0	1.2
Hallberg, Hallberg, and Kramer (2008)	0.0	2.0	2.0	0.0	0.0	2.0	1.0
Scarinci, Worrall, and Hickson (2009c)	0.0	2.0	2.0	2.0	2.0	2.0	1.7
McNeil, Gulliver, Morris, and Bance (2011)	0.0	0.0	1.0	2.0	2.0	2.0	1.2
Scarinci, Worrall, and Hickson (2012)	0.0	2.0	2.0	1.0	2.0	2.0	1.5
Preminger and Meeks (2012) (Study 1)	0.0	2.0	2.0	0.0	2.0	2.0	1.3
Preminger and Meeks (2012) (Study 2)	0.0	2.0	2.0	0.0	2.0	2.0	1.3
Zekveld, George, Houtgast, and Kramer (2013)	0.0	2.0	2.0	2.0	2.0	2.0	1.7
Govender et al. (2014)	0.0	1.0	1.0	2.0	1.0	2.0	1.2
Senkal, Kose, and Aksoy (2014)	0.0	2.0	2.0	2.0	2.0	0.0	1.3
Schulz et al. (2016)	2.0	2.0	2.0	2.0	0.0	2.0	1.7
Schulz et al. (2017)	2.0	2.0	2.0	2.0	2.0	2.0	2.0
**Mean score per item**	**0.1**	**1.5**	**1.8**	**0.9**	**0.6**	**1.1**	**1.0**

## Discussion

The primary objective of our review was to collect and synthesize generic and hearing-specific complaints in everyday life that are reported by people with hearing loss and their communication partners. After extensive searching, we found 78 eligible studies. Information presented across these studies broadly encapsulated auditory and nonauditory complaints due to hearing loss. These complaints were extracted from the studies then organized as two hierarchical frameworks, DoHL-P and DoHL-CP, each comprising the same three supra-domains (Auditory, Social, and Self).

Across both frameworks, there were 14 domains and 79 subdomains representing the impact of hearing loss. There was considerable overlap in some of these reported across both frameworks. Many of the subdomains within “Auditory: communicating” were experienced by both individuals with hearing loss and communication partners. For example, individuals with hearing loss reported difficulties participating in conversation in several different situations, which in turn had impacted on the communication partner. Another subdomain in the DoHL-P was difficulty hearing the telephone ring and listening to speech on the telephone. This was represented conversely in the DoHL-CP within “Self: role of communication partner” domain as “having to answer the telephone” and “having to say that phone is ringing.” This domain overall comprises the accommodations or additional roles communication partners have had to undertake as a result of their partner’s hearing loss. It represents the ways in which hearing loss has multifaceted effects on those close to a PHI, rather than purely at an individual level of someone experiencing hearing difficulties. Across domains that were common to both frameworks, there was some variability within the subdomains: For example, the subdomains within “Social: relationships” of the DoHL-P from that of the DoHL-CP. For people with hearing loss, this was not acknowledged apart from the effects of hearing loss on relationships with communication partners and family members in general. Within the domain “Social: social life,” communication partners reported that as a result of the negative experiences encountered by people with hearing loss, they experienced a reduced enjoyment of social activities and attending social events alone. This was also represented in the “sense of isolation” subdomains where communication partners reported feeling isolated as a couple at social events and a reduction in attending events as a couple. This ties in with the PHI domains of the subdomain of social withdrawal.

There were considerably more subdomains reported in the “Self: emotions” domain by partners. Communication partners reported the burden and stress of having to adjust to their partner’s hearing loss as well as the emotional consequences hearing loss imposed on the relationship with their partner. Communication partners reported feelings of guilt and upset in relation to the way they reacted to hearing loss and their lack of understanding of the PHI’s difficulties. Furthermore, communication partners reported far more subdomains within the “Self: effort and fatigue” domain. Effort in this context was particularly associated with having to accommodate and additional responsibilities and strategies undertaken to adapt to hearing loss. It is noteworthy that fatigue in relation to communication is present in both perspectives rather than the person experiencing hearing loss alone.

The “Auditory: communicating” domain contained the greatest overlap between patients and communication partners. This was particularly true for subdomains relating to conversation in different situations such as in noise or among a group of talkers. There was also some overlap in the subdomains within “emotions” such as the frustration, anger, and the upset of coping and living with hearing loss across patients and communication partners. In addition, self-image was a subdomain present in both frameworks. For communication partners, this was in relation to the image of themselves as a couple in society following hearing loss, as well as the image of the individual with hearing loss alone. Feelings of fatigue, in relation to listening and participating in conversation, were also reported by individuals with hearing loss and communication partners which again the shared problems hearing loss has on communication which involves interaction with other people.

Perhaps unsurprisingly, most of the subdomains in the “Auditory” supra-domain were unique to people with hearing loss, particularly those associated with listening and hearing sounds. In addition, the occupational impact of hearing loss on those experiencing hearing difficulties is only present in DoHL-P. These subdomains did not appear in DoHL-CP.

We presume that the overlap across the two frameworks reflects different manifestations of the same domain: that is, how a particular difficulty of someone with hearing loss can have a secondary impact on their communication partner. Audiological assessments in clinic emphasize the hearing status of an individual. Many clinical measures do not adequately capture the psychosocial consequences of hearing loss from both perspectives, and they are not typically aimed at communication partners. Considering the challenges encountered by communication partners as a result of hearing loss may contribute a more complete clinical profile of a patient undergoing audiological assessment and help optimize rehabilitation outcomes. Future research should thus explore ways in which these domains can be measured in the laboratory in order to integrate a holistic approach to clinical assessment of hearing loss: the DoHL-P and DoHL-CP can be used to guide the development of such measures.

We found in the survey that 19 different questionnaires were used. We mapped their individual items onto the domains and subdomains of the DoHL-P; see Supplementary file F (those domains that are not marked there were found in the qualitative literature only). The resulting distribution is quite uneven. About 20% to 30% of the possible cells within the Auditory: hearing, Auditory: listening, and Self: emotions domains are represented by items, but only about 15% of the Auditory: communication and Self: identity domains, and just 5% or less of Auditory: Speaking, Self: Effort & Fatigue, and Self: Stigma. Some subdomains (e.g., “listening to birdsong”, “reduced spontaneous conversation”) were entirely unrepresented. No single questionnaire asks about every domain: the closest is the Hearing-Dependent Daily Activities Scale (Hidalgo et al., 2008) with seven mapped questionnaire items. In the clinic, therefore, the full frameworks can only be explored with a battery of questionnaires or utilization of a questionnaire together with open-format questions.

Taken together, all these areas form part of a patient’s concerns that need be considered and factored into rehabilitation based on the patient-centered care model. This is a well-established component of the delivery of many health and rehabilitation interventions (Mead & Bower, 2000); yet, despite being suggestsed for adult aural rehabilitation, it is not yet adopted (Grenness, Hickson, Laplante-Levesque, & Davidson, 2014a, 2014b; Laplante-Lévesque, Hickson, & Worrall, 2012).

### Comparison With Other Studies

Hearing loss is a chronic condition that affects the whole family. Yet, to our knowledge, our work represents the first endeavor to create empirically derived frameworks of hearing loss complaints from literature that explores the perspective of people with hearing loss *and* their communication partners. Evidence from video-recorded audiology appointments indicates that family members have a strong interest in being involved and sharing their experiences of the patient’s hearing loss, but that they are typically discounted by the audiologist (Ekberg, Meyer, Scarinci, Grenness, & Hickson, 2015). Interestingly, some behaviors observed in these analyses showed how family members sometimes self-selected to speak in the appointment by responding to audiologist questions directed at the patient. This behavioral observation fits well with one of our subdomains, “having to act as an interpreter,” whereby communication partners described their role in speaking on behalf of the PHI. In another video study, Ekberg et al. (2014) observed many closed-form questions about medical and lifestyle issues, but there was very little emotionally focused conversation. From the same dataset, Ekberg et al. (2015) observed that patient-expressed concerns often conveyed their negative emotions, but that such concerns were not always adequately addressed by the audiologist.

Kamil and Lin (2015) conducted a systematic review to gain insight into the effects of hearing loss on the communication partners of older adults (≥50 years old) who are hard of hearing. The authors aimed to describe the effects of hearing impairment on communication partners of those with hearing loss. Similar to our findings, the authors found that the design of the included studies that investigated the impact of hearing loss on communication partners was varied, with some studies using qualitative methods such as interviews to gather information about the effects of hearing loss (Kelly & Atcherson, 2011; Scarinci et al., 2009a; Scarinci et al., 2009b) or open-ended surveys (Lormore & Stephens, 1994; Stephens et al., 1995). Other studies used quantitative methods such as questionnaires. In terms of hearing assessment, Kamil and Lin (2015) also found that the study participant’s hearing was tested and reported using a number of different methods such as pure-tone audiometry, speech recognition tests, and participant self-report. Due to the heterogeneity across studies, a meta-analysis could not be carried out. This also meant that the authors were unable to draw conclusions about the differences between those with hearing loss and communication partners. However, the authors report general findings regarding the role of communication partners in terms of caregiver burden and becoming an interpreter for the person with hearing loss. The impact of hearing loss on the relationship of the communication partner with someone who has hearing loss, the emotional consequences, and impact on social life are other findings reported. These are congruent with our findings.

A second prior review also gave findings consistent with ours. Barker et al. (2017) found that the psychosocial effects of hearing loss such as the negative associations of hearing loss with old age affected persons with hearing loss and communication partners and therefore acknowledge these experiences to be linked. Barker et al. (2017) also discussed the potential implications of the views held by the communication partner. A communication partner’s perception of the stigma of hearing aids could subtly alter the PHI’s decision to get a hearing aid. Altered self-image was noted from both person’s perspective, and the communication partner’s strategy of coping was often linked to the idea of projecting a “normal” image of the couple to other people. However, the authors search strategy targeted only those qualitative studies exploring the psychosocial experiences of hearing loss.

The field of health psychology provides a number of alternative frameworks for understanding a long-term health condition and its personal impact. Heffernan, Coulson, Henshaw, Barry, and Ferguson (2016) recently described the application of Leventhal’s self-regulatory model (Leventhal et al., 1997). This model proposes that the ways in which a person construes their own health condition affects the way that they cope with it and so ultimately their health outcomes. Important mediating components are thoughts and beliefs, and the emotional reactions to the condition. Interviews with 25 people with hearing loss explored these components of the self-regulatory model observing that “Most individuals with hearing loss reported negative emotional representations of hearing loss” (Heffernan et al., 2016, p. 6). Expressed emotions included frustration, irritation, embarrassment, and loneliness, which are captured as subdomains in our own inductive domain-grouping frameworks. Initial comparisons suggest that our domain groupings are congruent with the self-regulatory framework and the subdomains identified from the data-driven analysis could easily fit into this theoretically motivated framework. For example, aspects of “disengaged coping” mechanisms are captured here as “Social withdrawal,” “Pretending to understand speech,” and “Effort of having to act as an interpreter.” Further work, beyond the scope of the present synthesis, is needed to decide this.

The mean age of study participants across the included studies was generally poorly reported, particularly across the qualitative studies. For studies that did report age, the mean was 66.7 years. The UK Time Use Survey (2003) describes leisure choices made by older people. People above 65 years old tend to spend more time doing sedate activities such as watching TV and listening to music (Soule, 2005). “Successful aging” has been posed to feature social engagement, an aspect highlighted by our frameworks. This comprises “remaining involved in activities that are meaningful and purposeful” and “maintaining close relationships” (Adam, Leibbrandt, & Moon, 2011). Good listening and communicating ability is essential in order to participate in such activities. Giummarra, Haralambous, Moore, and Nankervis (2007) interviewed older people to gain insight on the relation between aging and social isolation. The authors found that older people reported social connectedness and social activity to be strongly associated with overall health. These components also form aspects of life that appear in our frameworks, such as social life and relationships that represent the negative implications of hearing loss.

### Strengths and Limitations of the Study

Our frameworks identify what experiences of living with hearing loss are shared and what are unique to each perspective. The included studies demonstrated considerable variation in the study population characteristics and in the degree and etiology of hearing loss among participants. Many studies did not fully describe the hearing status of the people with hearing loss and rarely if ever disclosed the hearing status of the communication partners. There may be cases where the communication partner may have an undiagnosed hearing loss themselves, and some of their own hearing difficulties might unknowingly contribute to the experiences described here. These limitations make it difficult to investigate how hearing status might influence the type of experiences and difficulties associated with hearing loss, but they also make it difficult to draw generalizable conclusions. It is also important to note that we did not find any data pertaining to communication partners who were not spouses or partners; there is nothing in the present literature about impacts on other communication partners such as siblings, children, friends, relatives, colleagues, and carers (Manchaiah et al., 2012). Given that the interest in communication partners is a relatively newer field of research, this was represented in the comparatively fewer data items extracted pertaining to this perspective compared with the person with hearing loss data. Having more data regarding the problems associated with loss from the perspective of communication partners who are friends or family members of the person with hearing loss may give further items to the DoHL-CP.

While the derivation of these domains consisted of a rigorous synthesis of a large amount of data, the domains and subdomains only represent hearing loss complaints that were reported by persons with hearing loss or communication partners in the studies analyzed. Every domain or subdomain could therefore only have entered into the framework either by someone mentioning it in response to an open query or because there was a corresponding item or subscale in a questionnaire. The questionnaires extracted in the review tapped into the subdomains depicted in the frameworks to varying degrees. The breadth can be illustrated by three examples: all three supra-domains are represented in the HHIE (Ventry & Weinstein, 1982), but only the Auditory: Hearing subdomain is represented in the Your Hearing questionnaire (Slawinski, Hartel, & Kline, 1993), and very few closed-set questionnaire items concern issues relating to “Self: identity.” None of the questionnaires analyzed here tapped into the subdomains of communicating at work, pretending to understand speech, stigma of hearing loss, and stigma of hearing aids.

There were a number of advantages to taking a data-driven approach in developing the frameworks, the primary one being that the frameworks are representative of patient- and communication partner-reported complaints, with minimized researcher bias. This does mean, however, that any complaints of hearing loss that were not reported in the included studies do not appear in the frameworks. The constructs represented in the each framework were not based on the frequency of reporting in the literature, other than the construct being mentioned at least once within the extracted data. All the domains and subdomains in the framework are therefore equally weighted. What remains unknown is whether that equivalence in weighting is a conceptual reality: Do people with hearing loss and their communication partners actually consider certain domains to play a more significant role in the daily lives? And to what extent are those impacts common to most people? This is a question for further research.

## Conclusions

Currently, auditory rehabilitation primarily serves to address the auditory degradation of hearing loss. However, the consequences of hearing loss are multifaceted and can extend to various aspects of life as well as on people close to those with hearing loss. The DoHL-P and DoHL-CP frameworks translate and summarize the vast qualitative research evidence of complaints of hearing loss into an evidence-based hierarchy. The data demonstrate that these complaints are Auditory (hearing, listening, communicating, speaking), Social (relationships, isolation, social life, occupational, interventions), and Self (effort and fatigue, emotions, identity, and stigma). These frameworks highlight aspects of hearing loss that are not currently addressed in currently aural rehabilitation plans, especially the far-reaching effects of hearing loss that may extend beyond the patient, particularly the effects on family members and their involvement the patient’s experience living with hearing loss. This is particularly important during the early stages of auditory assessment and diagnosis in order to form complete a clinical profile of patients with hearing loss and facilitate personalizing rehabilitation plans to consider the patient in the wider context of their circumstances.

## Supplementary Material

Supplementary material

Supplementary material

Supplementary material

Supplementary material

Supplementary material

Supplementary material
