# Supplementary material for: A Data-Driven Synthesis of Research Evidence for Domains of Hearing Loss, as Reported by Adults With Hearing Loss and Their Communication Partners
Source: Trends Hear. 2017 Oct 5;21:2331216517734088. doi: 10.1177/2331216517734088 (PMC5638151; doi:10.1177/2331216517734088)
Supplement: Supplementary material [file Supplementary_fileA.pdf]

Supplementary file A– Guidance for Data Collection

General Information

Primary research question: "What are the domains reported by people with hearing loss and their communication partner(s) which correspond to the aspects of hearing loss that are a problem for them?"

Category	Field	Guidance
GENERAL INFORMATION	**Researcher**	Initials of researcher performing the data collection
	**Publication number**	This is the code number given to the record to uniquely identify the study. The first record will be numbered SR_001 each record extracted will be numbered sequentially following the preceding article.
	**Author (Last name, first name)**	All author's names must be stated
	**Title of the study**	This is the full title of the record as given by the authors.
	**Type of publication**	Select from: Journal article, report, conference paper, book chapter.
	**Year of publication**	Paper-publication data of record (not date of submission or acceptance of record)
	**Country of Origin**	Specify country where the study was conducted.
STUDY CHARACTERISTICS	**Study Design**	Select from: Treatment, Observational, Cross-sectional, Exploratory, Other (Please specify in free-field text (cell AN in excel spreadsheet).
	**Is hearing the primary condition of interest?**	Select Yes/No to indicate whether focus of record is reported-complaints is in relation to hearing loss.
	**Primary method for collecting individual hearing loss complaints**	Select Questionnaire, Focus Group, Interview.
	**Specific questions asked**	If method of obtaining complaints associated with hearing loss is an interview format, state questions asked to participants to attain information.
	**Type of questions**	Select from: open, closed, open and closed, not stated.
	**Sample size (N analysed)**	State number of participants analysed in final results (also state number of male/participants)
	**Theoretical framework**	If indicated, specify theoretical framework underpinning rationale of study
PARTICIPANT CHARACTERISTICS	**Mean age**	Specify mean age of participants included in study. If not stated, extract information regarding inclusive age (range) from inclusion/exclusion criteria.
	**Age Range (m)**	Age range of female participants
	**Gender**	Specify gender of participants. Select from male, female, male and female, not stated.

	**Setting**	Select from general public, non-clinical, occupational group, primary care service (GP practices), secondary care service (audiology/ hospital clinics), and residential care (nursing homes).
	**Hearing Status**	Including mean audiometric thresholds, description of hearing loss severity, etiology of hearing loss.
HEARING LOSS COMPLAINTS	**Patient domains**	This refers to the dimension of hearing loss the authors intend to measure. Provide wording given by the authors or re-phrase to appropriately reflect domain. Specify every identified domain in a new cell.
	**Patient measures**	This refers to the method used to obtain domains. If questionnaire, specify the name (and reference). If interview/focus group, specify the questions asked to participants if provided. Specify each measure in new cell adjacent to the domain of interest.
	**Examples of patient domains**	Provide quotes of the most commonly and/or most significant complaints of hearing loss stated in record. If not provided in the record, refer to results section and extract the complaints the authors identify as the most commonly occurring. If the complaints are not explicit, then the researcher conducting the data extraction must interpret and convey the information to reflect the corresponding domain.
	**Perspective**	Specify who complaint or domain of hearing loss is referring to. Specify whether complaint refers to “self” or refers to communication partner.
	**Communication partner domains**	Provide quotes of the most commonly and/or most significant complaints of hearing loss. If not provided in the record, refer to results section and extract the complaints the authors identify as the most commonly occurring. If the complaints are not explicit, then the researcher conducting the data extraction must interpret and convey the information to reflect the corresponding domain.
	**Communication partner measures**	This refers to the method used to obtain domains. If questionnaire, specify the name (and reference). If interview/focus group, specify the questions (column L) asked to participants if provided.
	**Examples of communication partner domains**	Provide quotes of the most commonly and/or most significant complaints of hearing loss. If not provided in the record, refer to results section and extract the complaints the authors identify as the most commonly occurring. If the complaints are not explicit, then the researcher conducting the data extraction must interpret and convey the information to reflect the corresponding domain.

	**Perspective**	Specify who complaint or domain of hearing loss is referring to. Specify whether complaint refers to "self" or refers to communication partner.

Notes

Generated by Venessa Vas Friday 2nd October 2015
Amended by Deborah A. Hall and Venessa Vas Thursday 15th October 2015
