# Supplementary material for: A Data-Driven Synthesis of Research Evidence for Domains of Hearing Loss, as Reported by Adults With Hearing Loss and Their Communication Partners
Source: Trends Hear. 2017 Oct 5;21:2331216517734088. doi: 10.1177/2331216517734088 (PMC5638151; doi:10.1177/2331216517734088)
Supplement: Supplementary material [file Supplementary_fileB.pdf]

Supplementary file B: Miscellaneous items that could not be coded into domains

Domain/Complaint	Example
Environmental factors cause of difficulties	No example given
Self-rating handicap in working life	No example given
Noise at work	No example given
After a day at work, the hearing impaired person has the sensation that his ears are blocked	No example given
Travelling is difficult	No example given
Public services	Churches, banks, stores, government services, health services
Cinemas/ theatres/ chapels	No example given
Live speech	No example
One-sided speech	No example given
Temporal cues of speech	No example given
Communication is disrupted	"The communication is less personal"
Telephone marketing/sales	No example given
Meeting unfamiliar people	No example
Awareness of a hearing problem	Communication difficulties, Reactions to notification of these difficulties,
Tinnitus is very annoying	No example given
School playground duty	No example given
Public incidents	No example given
Dependence	No example given
Mobility	No example given
Maladaptive behaviour	No example given
Domestic life	No example given
Psychosocial	No example given
Learning and applying knowledge	No example given
Medical	No example given
General tasks and demands	No example given
Major life areas	No example given
Recreational providers reluctant to adapt activities to suit their needs	No example given
Medical	No example given
Activity limitation	No example given
Help-seeking	No example given
Background noise	"I am a very sensitive person when it comes to noise. I don't like noise…anywhere"
Physical or acoustical factors interfering	No example given
Background noise	No example given
Affected participation in daily life activities	No example given
Emotional withdrawal	No example given
Paranoid feelings	"I am often blamed for things that are just not my fault"
Blame	No example given
Difficulty in communicating the problem to clinician	No example given
Difficulty informing friends and co-workers about hearing loss	No example given
Decreased communicative performance	No example given

Supplementary file B: Miscellaneous items that could not be coded into domains
