# Supplementary material for: A Data-Driven Synthesis of Research Evidence for Domains of Hearing Loss, as Reported by Adults With Hearing Loss and Their Communication Partners
Source: Trends Hear. 2017 Oct 5;21:2331216517734088. doi: 10.1177/2331216517734088 (PMC5638151; doi:10.1177/2331216517734088)
Supplement: Supplementary material [file Supplementary_fileC.pdf]

Supplementary material 4: Tables reporting full reference list for all 78 included records.

Table 1: Reference list of included records that investigated the impact of hearing loss on persons with hearing loss only:

Record Number	Reference
SR_001	Cox RM, Alexander GC, Gray GA. Personality, hearing problems, and amplification characteristics: Contributions to self-report hearing aid outcomes. Ear and Hearing 2007;28(2):141-62
SR_002	Anderson DL, Noble W. Couples' attributions about behaviours modulated by hearing impairment: links with relationship satisfaction. International journal of audiology 2005;44(4):197-205
SR_019	Newman CW, Jacobson GP, Hug GA, et al. Perceived hearing handicap of patients with unilateral or mild hearing loss. The Annals of otology, rhinology, and laryngology 1997;106(3):210-4
SR_005	Garstecki DC. Self-perceived hearing difficulty in aging adults with acquired hearing loss. Journal of the Academy of Rehabilitative Audiology 1987;20:49-60
SR_006	Kelly RJ, Atcherson SR. Quality of life for individuals with hearing impairment who have not consulted for services and their significant others: same- and different-sex couples. Journal of communication disorders 2011;44(3):336-44 doi: http://dx.doi.org/10.1016/j.jcomdis.2011.01.004[published Online First: Epub Date]|
SR_007	Jonsson I, Hedelin B. Striving for genuine connections: men's experiences of living with hearing impairment. Scandinavian Journal of Disability Research 2012;14(4):313-26
SR_008	Hetu R, Lalonde M, Getty L. Psychosocial disadvantages associated with occupational hearing loss as experienced in the family. Audiology 1987;26(3):141-52
SR_009	Tesch-Römer C. Psychological effects of hearing aid use in older adults. The Journals of Gerontology Series B: Psychological Sciences and Social Sciences 1997;52(3):P127-P38
SR_012	Hass-Slavin L, McColl MA, Pickett W. Challenges and strategies related to hearing loss among dairy farmers. Journal of Rural Health 2005;21(4):329-36
SR_013	Tsuruoka H, Masuda S, Ukai K, et al. Hearing impairment and quality of life for the elderly in nursing homes. Auris Nasus Larynx 2001;28(1):45-54 doi: 10.1016/s0385-8146(00)00074-2[published Online First: Epub Date]|.
SR_035	Kochkin S, Rogin CM. Quantifying the obvious: the impact of hearing instruments on quality of life. Hear Rev 2000;7(1):6-34
SR_037	Ekberg K, Grenness C, Hickson L. Addressing Patients' Psychosocial Concerns Regarding Hearing Aids Within Audiology Appointments for Older Adults. American Journal of Audiology 2014;23(3):337-50 doi: 10.1044/2014[published Online First: Epub Date]
SR_036	Hidalgo JLT, Gras CB, Lapeira JMT, et al. The hearing-dependent daily activities scale to evaluate impact of hearing loss in older people. Annals of Family Medicine 2008;6(5):441-47
SR_039	Saunders GH and Forsline A. The Performance-Perceptual Test (PPT) and Its application to hearing aid counselling, The Hearing Review. (2006).

Supplementary material 4: Tables reporting full reference list for all 78 included records.

SR_051	Hallam R, Ashton P, Sherbourne K, et al. Persons with acquired profound hearing loss (APHL): how do they and their families adapt to the challenge? Health (London, England : 1997) 2008;12(3):369-88 doi: 10.1177/1363459308090054[published Online First: Epub Date]
SR_056	Claesen E, Pryce H. An exploration of the perspectives of help-seekers prescribed hearing aids. Primary health care research & development 2012;13(3):279-84 doi: 10.1017/s1463423611000570[published Online First: Epub Date]
SR_059	Gething L. Ageing with long-standing hearing impairment and deafness. International journal of rehabilitation research. Internationale Zeitschrift fur Rehabilitationsforschung. Revue internationale de recherches de readaptation 2000;23(3):209-15.
SR_066	Stephens SDG, Lewis PA, Charny MC, et al. CHARACTERISTICS OF SELF-REPORTED HEARING PROBLEMS IN A COMMUNITY SURVEY. Audiology 1990;29(2):93-100.
SR_072	Leposavić L, Leposavić I, Jasović-Gasić M, et al. Psychosocial aspects of acquired hearing impairment in the patients with otosclerosis. Psychiatria Danubina 2006;18(1-2):30-38 .
SR_074	Vesterager V, Salomon G, Jagd M. AGE-RELATED HEARING DIFFICULTIES .2. PSYCHOLOGICAL AND SOCIOLOGICAL CONSEQUENCES OF HEARING PROBLEMS - A CONTROLLED-STUDY. Audiology 1988;27(3):179-92.
SR_077	Martin FN, Krall L, O'Neal J. The diagnosis of acquired hearing loss. Patient reactions. ASHA 1989;31(11):47-50.
SR_079	Magilvy JK. Experiencing hearing loss in later life: a comparison of deaf and hearing-impaired older women. Research in nursing & health 1985;8(4):347-53.
SR_080	Hallberg, L. R. M., Hallberg, U., & Kramer, S. E. (2008). Self-reported hearing difficulties, communication strategies and psychological general well-being (quality of life) in patients with acquired hearing impairment. *Disability and rehabilitation*, *30*(3), 203-212.
SR_085	Mulrow CD, Aguilar C, Endicott JE, et al. QUALITY-OF-LIFE CHANGES AND HEARING IMPAIRMENT - A RANDOMIZED TRIAL. Annals of Internal Medicine 1990;113(3):188-94.
SR_090	Miyakita T, Ueda A, Zusho H, et al. Self-evaluation scores of hearing difficulties and quality of life components among retired workers with noise-related hearing loss. Journal of Sound and Vibration 2002;250(1):119-28 doi: 10.1006/jsvi.2001.3898[published Online First: Epub Date.
SR_091	Stark P, Hickson L. Outcomes of hearing aid fitting for older people with hearing impairment and their significant others. International Journal of Audiology 2004;43(7):390-98.
SR_097	Slawinski EB, Hartel DM, Kline DW. Self-reported hearing problems in daily life throughout adulthood. Psychology and aging 1993;8(4):552-61.
SR_098	Helvik A-S, Wennberg S, Jacobsen G, et al. Coping ability and everyday life situations in relation to audiological rehabilitation. Audiological Medicine 2007;5(2):112-18.
SR_102	Preminger JE, Laplante-Levesque A. Perceptions of age and brain in relation to hearing help-seeking and rehabilitation. Ear and hearing 2014;35(1):19-29 doi: 10.1097/AUD.0b013e31829c065c[published Online First: Epub Date].
SR_103	Bade P. Hearing impairment and the elderly patient. Wisconsin medical journal 1991;90(9):516-19
SR_104	Espmark AK, Rosenhall U, Erlandsson S, et al. The two faces of presbyacusis: hearing impairment and psychosocial consequences. International Journal of Audiology 2002;41(2):125-35.
SR_105	Hallam RS, Brooks DN. Development of the Hearing Attitudes in Rehabilitation Questionnaire (HARQ). British journal of audiology 1996;30(3):199-213.

Supplementary material 4: Tables reporting full reference list for all 78 included records.

SR_106	Strawbridge WJ, Wallhagen MI, Shema SJ, et al. Negative consequences of hearing impairment in old age: a longitudinal analysis. Gerontologist 2000;40(3):320-6.
SR_107	Laroche C, Garcia LJ, Barrette J. Perceptions by persons with hearing impairment, audiologists, and employers of the obstacles to work integration. Journal of the Academy of Rehabilitative Audiology 2000;33:63-90.
SR_110	Plath P. Problems in fitting hearing aids in the elderly. Acta oto-laryngologica. Supplementum 1990;476:278-80.
SR_118	Akin Senkal O, Kose A and Aksoy S. Assessment of geriatric patients' satisfaction on hearing aids and their influence on quality of life. Turkish Journal of Geriatrics 2014; 17 (4) 389-396.
SR_122	Punch R, Hyde M, Power D. Career and workplace experiences of Australian university graduates who are deaf or hard of hearing. Journal of deaf studies and deaf education 2007;12(4):504-17 doi: 10.1093/deafed/enm011[published Online First: Epub Date].
SR_127	Zekveld AA, George EL, Houtgast T, et al. Cognitive abilities relate to self-reported hearing disability. Journal of speech, language, and hearing research : JSLHR 2013;56(5):1364-72 doi: 10.1044/1092-4388(2013/12-0268)[published Online First: Epub Date].
SR_134	Granberg S, Pronk M, Swanepoel DW, et al. The ICF core sets for hearing loss project: Functioning and disability from the patient perspective. International Journal of Audiology 2014;53(11):777-86 doi: 10.3109/14992027.2014.938370[published Online First: Epub Date].
SR_140	Jennings MB, Shawb L. Impact of hearing loss in the workplace: Raising questions about partnerships with professionals. Work 2008;30(3):289-95
SR_144	Mulrow CD, Aguilar C, Endicott JE, et al. association between hearing impairment and the quality of life of elderly individuals. Journal of the American Geriatrics Society 1990;38(1):45-50.
SR_161	Vesterager V, Salomon G. Psychosocial aspects of hearing impairment in the elderly. Acta oto-laryngologica. Supplementum 1990;476:215-20.
SR_164	Caissie R, Gibson CL. The effectiveness of repair strategies used by people with hearing losses and their conversational partners. Volta Review 1997;99(4):203-18.
SR_168	Albera R, Cavalot A, II, Beatrice F, et al. Self-reported disability and handicap in individuals with noise-induced hearing loss. Journal of Audiological Medicine 2001;10(3):175-83.
SR_172	Gilbertson M, Fusilier B, Murch S, et al. Speech concerns of hard-of-hearing adults. Perceptual Motor Skills 1996;83(2):377-8 doi: 10.2466/pms.1996.83.2.377[published Online First: Epub Date]
SR_173	Vuorialho, A., Karinen, P., & Sorri, M. (2006). Effect of hearing aids on hearing disability and quality of life in the elderly: Efecto de los auxiliares auditivos (AA) en la discapacidad auditiva y la calidad de vida de los ancianos. *International Journal of Audiology*, *45*(7), 400-405.
SR_211	Cowie R, Watson D, Kerr P, et al. Psychology and hearing impairment: Focussing on the people with the loss. Irish Journal of Psychology 1995;16(4):288-98.
SR_217	Gatehouse S. Glasgow Hearing Aid Benefit Profile: Derivation and Validation of. Journal of the American Academy of Audiology 1999;10:80-103.
SR_226	Heffernan, E., Coulson, N. S., Henshaw, H., Barry, J. G., & Ferguson, M. A. (2016). Understanding the psychosocial experiences of adults with mild-moderate hearing loss: an application of Leventhal's self-regulatory model. *International journal of audiology*, *55*(sup3), S3-S12.

Supplementary material 4: Tables reporting full reference list for all 78 included records.

**Record Number**	**Reference**
SR_011	Govender NG, Maistry N, Soomar N, et al. Hearing loss within a marriage: Perceptions of the spouse with normal hearing. South African Family Practice 2014;56(1):50-56.
SR_033	Robinson J. and Humes M. Counseling the significant other in the hearing aid process. Hearing Journal. 2004; 3: 44-50.
SR_061	Hallberg, L. R. M. (1999). Hearing impairment, coping, and consequences on family life. *JOURNAL-ACADEMY OF REHABILITATIVE AUDIOLOGY*, *32*, 45-60.
SR_082	Manchaiah VK, Stephens D, Lunner T. Communication Partners' Journey through Their Partner's Hearing Impairment. Int J Otolaryngol 2013;2013:707910 doi: 10.1155/2013/707910[published Online First: Epub Date].
SR_109	Scarinci N, Worrall L, Hickson L. The effect of hearing impairment in older people on the spouse: development and psychometric testing of the significant other scale for hearing disability (SOS-HEAR). International journal of audiology 2009;48(10):671-83 doi: 10.1080/14992020902998409[published Online First: Epub Date].
SR_145	Preminger JE, Meeks S. The Hearing Impairment Impact-Significant Other Profile (HII-SOP): A Tool to Measure Hearing Loss-Related Quality of Life in Spouses of People with Hearing Loss. Journal of the American Academy of Audiology 2012;23(10):807-23 doi: 10.3766/jaaa.23.10.6[published Online First: Epub Date].
SR_150	Scarinci N, Worrall L, Hickson L. The effect of hearing impairment in older people on the spouse. International Journal of Audiology 2008;47(3):141-51.
SR_174	Scarinci N, Worrall L, Hickson L. The ICF and third-party disability: its application to spouses of older people with hearing impairment. Disability and rehabilitation 2009;31(25):2088-100 doi: 10.3109/09638280902927028[published Online First: Epub Date].
SR_188	McNeil ML, Gulliver M, Morris DP, et al. Quality of life improvement for bone-anchored hearing aid users and their partners. The Journal of laryngology and otology 2011;125(6):554-60 doi: 10.1017/s0022215111000557[published Online First: Epub Date].

Reference list of included records that investigated the impact of hearing loss on communication partners only:

Supplementary material 4: Tables reporting full reference list for all 78 included records.

Reference list of included records that investigated the impact of hearing loss on persons with hearing loss *and* communication partner:

Record Number	Reference
SR_087	Brooks DN, Hallam RS, Mellor PA. The effects on significant others of providing a hearing aid to the hearing-impaired partner. British journal of audiology 2001;35(3):165-71.
SR_088	Knutson JF, Lansing CR. The relationship between communication problems and psychological difficulties in persons with profound acquired hearing loss. The Journal of speech and hearing disorders 1990;55(4):656-64.
SR_116	Scarinci N, Worrall L, Hickson L. Factors associated with third-party disability in spouses of older people with hearing impairment. Ear and hearing 2012;33(6):698-708 doi: 10.1097/AUD.0b013e31825aab39[published Online First: Epub Date].
SR_119	Hallberg, L. R. M., & Barrenäs, M. L. (1993). Living with a male with noise-induced hearing loss: experiences from the perspective of spouses. *British journal of audiology*, *27*(4), 255-261.
SR_120	Wanstrom G, Oberg M, Rydberg E, et al. The psychological process from avoidance to acceptance in adults with acquired hearing impairment. Hearing, Balance and Communication 2014;12(1):27-35.
SR_123	Yorgason, J. B., Piercy, F. P., & Piercy, S. K. (2007). Acquired hearing impairment in older couple relationships: An exploration of couple resilience processes. *Journal of Aging Studies*, *21*(3), 215-228.
SR_135	Hallberg LR, Barrenas ML. Group rehabilitation of middle-aged males with noise-induced hearing loss and their spouses: evaluation of short- and long-term effects. British journal of audiology 1994;28(2):71-9.
SR_137	Hetu R, Getty L, Waridel S. Attitudes towards co-workers affected by occupational hearing loss II: Focus groups interviews. British Journal of Audiology 1994;28(6):313-25.
SR_138	Hetu R, Riverin L, Getty L, et al. The reluctance to acknowledge hearing difficulties among hearing-impaired workers. British Journal of Audiology 1990;24(4):265-76.
SR_139	Hetu R, Riverin L, Lalande N, et al. Qualitative analysis of the handicap associated with occupational hearing loss. British Journal of Audiology 1988;22(4):251-64.
SR_142	Lormore KA, Stephens SD. Use of the open-ended questionnaire with patients and their significant others. British journal of audiology 1994;28(2):81-9.
SR_154	Stephens D, France L, Lormore K. Effects of hearing impairment on the patient's family and friends. Acta Oto-Laryngologica 1995;115(2):165-67.
SR_159	Thiede KM. PSYCHOSOCIAL IMPACT OF HEARING LOSS IN THE ELDERLY AS REPORTED BY THE PATIENT AND A FAMILY MEMBER [M.S.N .]. Michigan State University, 1986.
SR_176	Morgan-Jones RA. *Hearing differently: the impact of hearing impairment on family life*: Whurr, 2001.
SR_206	Newman CW, Weinstein BE. Judgments of perceived hearing handicap by hearing-impaired elderly men and their spouses. Journal of the Academy of Rehabilitative Audiology 1986;19:109-15.

Supplementary material 4: Tables reporting full reference list for all 78 included records.

SR_210	Ross L, Lyon P. Escaping a silent world: Profound hearing loss, cochlear implants and household interaction. International Journal of Consumer Studies 2007;31(4):357-62.
SR_220	Wallhagen MI. The stigma of hearing loss. Gerontologist 2010;50(1):66-75 doi: 10.1093/geront/gnp107[published Online First: Epub Date].
SR_222	Morgan-Jones R. The impact of hearing impairment on the couple, the family and the social network [Ph.D.]. London School of Economics and Political Science (United Kingdom), 1998.
SR_223	Schulz, K. A., Modeste, N., Lee, J., Roberts, R., Saunders, G. H., & Witsell, D. L. (2016). Factors influencing pursuit of hearing evaluation: Enhancing the health belief model with perceived burden from hearing loss on communication partners. *International journal of audiology*, *55*(sup3), S69-S78.
SR_224	Schulz, K. A., Modeste, N., Lee, J. W., Roberts, R., Saunders, G. H., & Witsell, D. L. (2017). Burden of Hearing Loss on Communication Partners and Its Influence on Pursuit of Hearing Evaluation. *Ear and Hearing*.
