# Supplementary material for: A Data-Driven Synthesis of Research Evidence for Domains of Hearing Loss, as Reported by Adults With Hearing Loss and Their Communication Partners
Source: Trends Hear. 2017 Oct 5;21:2331216517734088. doi: 10.1177/2331216517734088 (PMC5638151; doi:10.1177/2331216517734088)
Supplement: Supplementary material [file Supplementary_fileD.pdf]

Supplementary file D-ICF Core-set data (components not covered)

Component-not-covered categories	Patient framework	Communication partner framework
s-nc mutations	–	–
d-nc waking up (as in waking up in the morning)	–	–
e-nc outdoor (outdoor in general as a context)	Hearing sounds	–
e-nc behavior of others (extremely frequent among all studies)	Social, Relationships, Occupational	–
d-nc participation (as in engaging in general activities)	Social interaction	Role of CP
e-nc other peoples skills and knowledge of HL	Occupational	Role of CP
d-nc activities (as in general activities)	–	Role of CP
e-nc home environment	–	Role of CP
e-nc visual cues	–	Role of CP
