# Supplementary material for: A Data-Driven Synthesis of Research Evidence for Domains of Hearing Loss, as Reported by Adults With Hearing Loss and Their Communication Partners
Source: Trends Hear. 2017 Oct 5;21:2331216517734088. doi: 10.1177/2331216517734088 (PMC5638151; doi:10.1177/2331216517734088)
Supplement: Supplementary material [file Supplementary_fileE.pdf]

Supplementary file E – Table showing how the individual complaints were grouped according to hearing loss severity

Mild Hearing Loss	Mild-moderate hearing loss	Moderate hearing loss
Negative social consequences of HI in everyday life	Casual beliefs	Social situations
Unable to hear non-verbal sounds in everyday life	Controllability/curability	Emotional consequences
Participation restriction	Emotional representations of hearing loss	"I understand that I am too cautious about my hearing. I am about everything"
Cannot hear when people ae speaking from a distance	Cognitive representations of hearing loss	"I don't have a good understanding of what hearing aids can and cannot do"
Communication problems		"I am not always aware of what I can hear"
Communication partner domains only		"I have more respect for the hearing aids and wear them more regularly now"
Stigma		"I feel as though hearing aids help me more than I originally thought they did"
Changes in speech since their hearing loss		"I need to think about what to expect from hearing aids"
Emotional problems		Frustration
Subjects felt handicapped because of their hearing loss		Detection difficulties
Hearing loss limits their lives		Feelings of inadequacy
Perceive hearing loss to be less handicapping than what spouse perceives		Problems with conversation
TV/Radio too loud		Feeling left out in group conversation
"The positive feedback you gave me about my hearing being better with my hearing aids than I thought was very helpful		Little enjoyment of free time
"I am willing to depend more on my hearing aids now"		Feeling lonely or remote
"You telling me about my hearing loss has helped me explain it to my wife and daughters"		Not feeling close to others
		Feeling left out in a group
		Noise at work
