# Supplementary material for: A Data-Driven Synthesis of Research Evidence for Domains of Hearing Loss, as Reported by Adults With Hearing Loss and Their Communication Partners
Source: Trends Hear. 2017 Oct 5;21:2331216517734088. doi: 10.1177/2331216517734088 (PMC5638151; doi:10.1177/2331216517734088)
Supplement: Supplementary material [file Supplementary_fileF.pdf]

Supplementary file F - Extracted questionnaire items (PHI) mapped on DoHL-P and DoHL-CP

	Hearing Handicap Inventory for the Elderly	Hearing Dependent Daily Activities Scale	Hearing Disabilities and Handicap Scale	Minnesota Multiphasic Personality Inventory	Your Hearing	Hearing Attitudes In Rehabilitation	Self-Evaluation of Self-function	McCarthy-Alpiner Scale of Hearing Handicap	Glasgow Hearing Aid Benefit Profile	Hearing and Communication Questionnaire	Quality of life questionnaire	Hearing Handicap Inventory for Elderly-Adult screening	Cardiff Health survey	Hearing Loss Diagnostic Experience Survey	Social, Emotional and Communication function	Self-assessed Hearing Questionnaire	Work Experience with Hearing Loss	Author created	Hearing Difficulties
**HEARING**																			
Hearing distant sounds		✓			✓				✓						✓	✓			
Hearing environmental sounds		✓			✓				✓	✓						✓			
Hearing speech sounds					✓			✓	✓				✓		✓	✓			
Hearing telephone ring			✓						✓	✓			✓			✓	✓		
Hearing television and radio sounds			✓							✓			✓		✓	✓	✓		
Hearing the doorbell			✓						✓										
Hearing warning sounds		✓			✓				✓	✓					✓	✓	✓	✓	
**LISTENING**																			
Listening to birdsong																			
Listening to music	✓	✓													✓	✓			
Listening to quiet speech (whispering)	✓	✓			✓														
Listening to speech (at work)	✓								✓										
Listening to speech (speech in general)	✓							✓	✓							✓			
Listening to speech in a group of talkers	✓	✓			✓	✓		✓	✓							✓			
Listening to speech in a travelling vehicle									✓							✓			
Listening to speech in public places	✓	✓						✓	✓				✓		✓	✓			
Listening to speech in quiet environments					✓				✓										
Listening to speech on the telephone		✓			✓				✓										
Pleasure of listening to music																✓			
Raising the volume of the television/radio	✓	✓	✓						✓	✓		✓	✓		✓	✓	✓		
**COMMUNICATING**																			
Asking people to repeat			✓																
Communicating at work																			
Communicating, general conversation	✓	✓	✓					✓	✓	✓			✓			✓			✓
Communicating, speech in quiet	✓	✓			✓			✓	✓	✓		✓				✓			
Conversing on the telephone	✓									✓							✓		
Reduced communication																			
Asking people to repeat										✓									
Communicating at work	✓								✓										
Communicating, general conversation		✓						✓	✓	✓		✓							
Communicating, speech in quiet		✓						✓	✓			✓							
Conversing in a group of talkers	✓	✓	✓					✓	✓										
Conversing in noisy environments	✓	✓	✓		✓			✓	✓				✓		✓				
Conversing one to one								✓											
Having to speak on behalf of their partner with hearing loss																			
Reduced spontaneous conversation																			
**SPEAKING**																			
Speech with a loud voice								✓											

Supplementary file F - Extracted questionnaire items (PHI) mapped on DoHL-P and DoHL-CP

	Hearing Handicap Inventory for the Elderly	Hearing Dependent Daily Activities Scale	Hearing Disabilities and Handicap Scale	Minnesota Multiphasic Personality Inventory	Your Hearing	Hearing Attitudes In Rehabilitation	Self-Evaluation of Self-function	McCarthy-Alpiner Scale of Hearing Handicap	Glasgow Hearing Aid Benefit Profile	Hearing and Communication Questionnaire	Quality of life questionnaire	Hearing Handicap Inventory for Elderly-Adult screening	Cardiff Health survey	Hearing Loss Diagnostic Experience Survey	Social, Emotional and Communication function	Self-assessed Hearing Questionnaire	Work Experience with Hearing Loss	Author-created	Hearing Difficulties
**INTERVENTIONS**																			
Expectations of rehabilitation						✓													
Technical problems with hearing aid						✓													
**ISOLATION**																			
Sense of isolation	✓			✓		✓		✓				✓							✓
**OCCUPATIONAL**																			
Fear for loss of employment														✓	✓	✓	✓	✓	✓
Reduced employment prospects														✓	✓	✓	✓	✓	✓
Opportunities at work														✓			✓	✓	✓
**RELATIONSHIPS**																			
Relationship with family members				✓				✓				✓					✓		
Relationship with spouse/family member			✓	✓				✓				✓					✓		
**SOCIAL LIFE**																			
Altered social interactions	✓		✓	✓		✓	✓	✓				✓					✓		
Social withdrawal	✓		✓	✓		✓	✓	✓				✓					✓		

Supplementary file F - Extracted questionnaire items (PHI) mapped on DoHL-P and DoHL-CP

	Hearing Handicap Inventory for the Elderly	Hearing Dependent Daily Activities Scale	Hearing Disabilities and Handicap Scale	Minnesota Multiphasic Personality Inventory	Your Hearing	Hearing Attitudes In Rehabilitation	Self-Evaluation of Self-function	McCarthy -Alpiner Scale of Hearing Handicap	Glasgow Hearing Aid Benefit Profile	Hearing and Communication Questionnaire	Quality of life questionnaire	Hearing Handicap Inventory for Elderly-Adult screening	Cardiff Health survey	Hearing Loss Diagnostic Experience Survey	Social, Emotional and Communication function	Self-assessed Hearing Questionnaire	Work Experience with Hearing Loss	Author-created	Hearing Difficulties
**EFFORT & FATIGUE**																			
Listening effort			✓																
Feelings of fatigue			✓																
**EMOTIONS**																			
Embarrassment	✓						✓			✓		✓		✓					
Rejection	✓		✓				✓			✓		✓							✓
Worry	✓						✓			✓		✓							✓
Anger	✓						✓			✓		✓							
Frustration	✓					✓	✓			✓		✓							
Upset	✓						✓			✓		✓							
**IDENTITY**																			
Feelings of inadequacy/self-esteem			✓			✓								✓					
Self-image			✓											✓					
**STIGMA**																			
Pretending to understand speech																			
Stigma of hearing aids																			
Denial																			
Stigma of hearing loss			✓			✓													

Table of references for questionnaires used in included studies:

**Questionnaire**	**Reference**
Hearing Handicap Inventory for the Elderly	(Ventry and Weinstein, 1982)
Hearing Dependent Daily Activities Scale	(Hidalgo et al., 2008)
Hearing Disabilities and Handicap Scale	(Miyakita et al., 2002)
Minnesota Multiphasic Personality Inventory	(Butcher, 2001)
Your Hearing	(Slawinski et al., 1993)
Hearing Attitudes in Rehabilitation Questionnaire	(Hallam and Brooks, 1996)
Self-Evaluation of Self-function	(Mulrow et al., 1990)
McCarthy-Alpiner Scale of Hearing Handicap	(Thiede, 1986)
Glasgow Hearing Aid Benefit Profile	(Gatehouse, 1999)
Hearing and Communication Questionnaire	(Knussen et al., 2004)
Quality of life questionnaire	(Tsuruoka et al., 2001)
Hearing Handicap Inventory for Elderly – Adult Screening	(Ventry and Weinstein, 1982)
Cardiff Health Survey	(Stephens et al., 1990)
Hearing Loss Diagnostic Experience Survey	(Martin et al., 1989)
Social, Emotional and Communication Function	(Bade, 1991)
Self-Assessed Hearing Questionnaire	(Espmark et al., 2002)
Work Experience with Hearing Loss	(Punch et al., 2007)
Author created (no name)	(Stephens et al., 1995)
Hearing Difficulties	(Cowie et al., 1995)
